# Cinacalcet in Patients with Chronic Kidney Disease: A Cumulative Meta-Analysis of Randomized Controlled Trials

**DOI:** 10.1371/journal.pmed.1001436

**Published:** 2013-04-30

**Authors:** Suetonia C. Palmer, Ionut Nistor, Jonathan C. Craig, Fabio Pellegrini, Piergiorgio Messa, Marcello Tonelli, Adrian Covic, Giovanni F. M. Strippoli

**Affiliations:** 1 Department of Medicine, University of Otago Christchurch, Christchurch, New Zealand; 2 Department of Nephrology, “Gr. T. Popa” University of Medicine and Pharmacy, Iasi, Romania; 3 School of Public Health, University of Sydney, Sydney, New South Wales, Australia; 4 Consorzio Mario Negri Sud, Santa Maria Imbaro, Italy; 5 Scientific Institute Casa Sollievo della Sofferenza, San Giovanni Rotondo, Italy; 6 Dialysis and Renal Transplant Unit, Department of Nephrology, Fondazione IRCCS Ca' Granda Ospedale Maggiore Policlinico, Milan, Italy; 7 University of Alberta, Edmonton, Alberta, Canada; 8 Diaverum Scientific Medical Office, Lund, Sweden; Yale University School of Medicine, United States of America

## Abstract

**Background:**

Calcimimetic agents lower serum parathyroid hormone levels in people with chronic kidney disease (CKD), but treatment effects on patient-relevant outcomes are uncertain. We conducted a systematic review and meta-analysis to summarize the benefits and harms of calcimimetic therapy in adults with CKD and used cumulative meta-analysis to identify how evidence for calcimimetic treatment has developed in this clinical setting.

**Methods and Findings:**

Cochrane and Embase databases (through February 7, 2013) were electronically searched to identify randomized trials evaluating effects of calcimimetic therapy on mortality and adverse events in adults with CKD. Two independent reviewers identified trials, extracted data, and assessed risk of bias.

Eighteen trials comprising 7,446 participants compared cinacalcet plus conventional therapy with placebo or no treatment plus conventional therapy in adults with CKD. In moderate- to high-quality evidence (based on Grading of Recommendations Assessment, Development, and Evaluation criteria) in adults with CKD stage 5D (dialysis), cinacalcet had little or no effect on all-cause mortality (relative risk, 0.97 [95% confidence interval, 0.89–1.05]), had imprecise effect on cardiovascular mortality (0.67 [0.16–2.87]), and prevented parathyroidectomy (0.49 [0.40–0.59]) and hypercalcemia (0.23 [0.05–0.97]), but increased hypocalcemia (6.98 [5.10–9.53]), nausea (2.02 [1.45–2.81]), and vomiting (1.97 [1.73–2.24]). Data for clinical outcomes were sparse in adults with CKD stages 3–5. On average, treating 1,000 people with CKD stage 5D for 1 y had no effect on survival and prevented about three patients from experiencing parathyroidectomy, whilst 60 experienced hypocalcemia and 150 experienced nausea. Analyses were limited by insufficient data in CKD stages 3–5 and kidney transplant recipients.

**Conclusions:**

Cinacalcet reduces the need for parathyroidectomy in patients with CKD stage 5D, but does not appear to improve all-cause or cardiovascular mortality. Additional trials in CKD stage 5D are unlikely to change our confidence in the treatment effects of cinacalcet in this population.

*Please see later in the article for the Editors' Summary*

## Abstract 2

Giovanni Strippoli and colleagues report findings of a systematic review and meta-analysis examining the benefits and harms of calcimimetic therapy in adults with chronic kidney disease.

## Editors' Summary

**Background:**

Chronic kidney disease (CKD) refers to a progressive loss in kidney function over time and is caused by several conditions such as diabetes and high blood pressure. CKD is an increasingly common condition, affecting an estimated 26 million adults in the United States alone, with millions more at risk. People with CKD are at increased risk of death compared to the general population, and despite recent improvements in care, people with advanced CKD who are receiving dialysis experience an excess mortality rate of up to 20%. People with CKD also experience several biochemical imbalances—such as increased phosphorus (as a result of retention), low vitamin D and calcium (hypocalcemia), and increased parathyroid hormone—which lead to further health problems (such as a thinning of the bones) and pain. In light of this complication, clinical guidelines for people with CKD currently recommend that the blood levels of phosphorus and parathyroid hormone be maintained in a certain range to prevent the need for surgical removal of the gland (surgical parathyroidectomy).

**Why Was This Study Done?:**

There are several medical therapies that help to keep phosphorus and parathyroid hormone blood levels within the target range, and in 2004, the drug cinacalcet hydrochloride (cinacalcet) was approved in the United States for use in people with end-stage CKD for this purpose. However, despite increasing use and costs of this drug (in the US, cinacalcet has become the largest single drug cost for patients on dialysis, with an annual expenditure of at least US$260 million, and in the UK, prescribing costs increased by up to 33% from 2010 to 2011), the therapeutic value of this drug remains uncertain. So in this study, the researchers systematically reviewed appropriate published studies and analyzed relevant data to investigate whether treatment with cinacalcet improves clinical outcomes in adults with CKD.

**What Did the Researchers Do and Find?:**

The researchers identified relevant randomized controlled trials conducted in participants with end-stage CKD by thoroughly searching electronic databases and trial registries, specialist journals, and major conference proceedings. They then extracted data according to specific criteria and assessed the risk of bias. The researchers then graded the quality of evidence into the categories high, moderate, low, and very low quality.

Using these techniques, the researchers included 18 trials involving 7,446 adults with CKD in their analysis. All included trials evaluated cinacalcet (at a dose of 30 mg to 180 mg/day) and how this drug compared to conventional therapy or conventional therapy plus placebo. The researchers found that, compared to placebo or no treatment, in high-quality evidence, cinacalcet had little or no effect on all-cause mortality (relative risk [RR], 0.97) for people in the final stage of CKD (stage 5D) and unclear effects on all-cause mortality in low-quality evidence for people in the less severe stages (stages 3–5) of CKD (RR, 0.29). Cinacalcet also had uncertain effects on cardiovascular mortality for participants with CKD stage 5D (RR, 0.67) and CKD stages 3–5 (RR, 0.29), and also its role in fracture prevention was unclear in people with CKD stage 5D. Furthermore, cinacalcet had significant side effects, such as increased risk of hypocalcemia (RR, 7.38), nausea (RR, 2.05), vomiting (RR, 1.95), and diarrhea (RR, 1.15), but reduced the risk of hypercalcemia (RR, 0.23). The researchers found that in high-quality evidence, cinacalcet prevented surgical parathyroidectomy in people with CKD stage 5D (RR, 0.49). However, overall, they also found that, on average, routinely treating 1,000 people with cinacalcet for one year has no effect on mortality, prevents about three people from experiencing surgical parathyroidectomy, and leads to approximately 60 and 150 people experiencing hypocalcemia and nausea, respectively.

**What Do These Findings Mean?:**

These findings suggest that routine cinacalcet (30 to 180 mg/day) therapy in people with end-stage CKD (stage 5D) reduces parathyroid concentrations and hypercalcemia, and may prevent surgical parathyroidectomy, but has little or no effect on all-cause mortality, has an unclear effect on cardiovascular death, and is frequently associated with adverse effects such as hypocalcemia, nausea, vomiting, and diarrhea—adverse gastrointestinal effects that may affect nutrition and quality of life. Although the evidence in people with CKD stages 3–5 is limited and generally of low quality, this analysis shows that despite the widespread use of cinacalcet and the associated high costs, this drug provides little clinical benefit. Therefore, routine use of cinacalcet therapy in people with CKD does not appear warranted; benefits may be limited to preventing parathyroidectomy in the small number of patients for whom surgery is contraindicated.

**Additional Information:**

Please access these websites via the online version of this summary at http://dx.doi.org/10.1371/journal.pmed.1001436.

- Wikipedia has information on the pharmacological profile of cinacalcet (note that Wikipedia is a free online encyclopedia that anyone can edit)
- The National Kidney Foundation has some useful information for patients with CKD, as does MedlinePlus

## Introduction

People with chronic kidney disease (CKD) [1] experience mortality in excess of the general population largely because of accelerated cardiovascular disease [2]–[4]. Although improvements in care have occurred, people with advanced CKD treated with dialysis (CKD stage 5D) experience annual mortality of approximately 15% to 20% [5]. Despite intensive efforts, numerous interventions to improve clinical outcomes in adults with CKD have failed to demonstrate beneficial effects on mortality or cardiovascular events, particularly for people treated with dialysis [6]–[10].

CKD leads progressively to phosphorus retention, impaired vitamin D activation, hypocalcemia, and increased parathyroid hormone (PTH) secretion. However, while global clinical practice guidelines suggest that serum PTH and phosphorus concentrations should be kept within a target range [11], therapies that ameliorate abnormal serum PTH and phosphorus levels (vitamin D compounds and phosphorus binders) have not been shown to improve clinical outcomes in randomized trials [12],[13]. In 2004, cinacalcet hydrochloride was approved in the United States to lower elevated serum PTH levels in patients with CKD stage 5D [14]. Cinacalcet mimics the action of calcium on calcium-sensing receptors in the parathyroid glands to suppress PTH secretion [15],[16] and, based on promising data, has been considered a potential intervention to prevent cardiovascular events and mortality in CKD [17],[18]. Within a decade of the first small randomized trials for cinacalcet [19],[20], and despite an earlier meta-analysis showing no evidence for benefit on clinical outcomes [21], cinacalcet prescribing has become the largest single drug cost for dialysis patients in the United States, with an annual expenditure of at least US$260 million, and community prescribing costs for cinacalcet in the United Kingdom increased by 20%–33% from 2010 to 2011 [5],[22]. A pooled analysis of four placebo-controlled randomized trials of cinacalcet in 2005 showing a large reduction in cardiovascular hospitalization with cinacalcet may have contributed to uncertainty in the medical community about the therapeutic benefits of cinacalcet therapy [18].

In light of widespread use and the recent publication of the largest randomized trial of cinacalcet in dialysis patients [23], we have conducted a systematic review and meta-analysis to summarize the available evidence that calcimimetic therapy improves clinical outcomes in adults with CKD. In the context of high cinacalcet prescribing costs despite an earlier meta-analysis reporting no evidence for benefit [21], we have used cumulative meta-analysis to evaluate the evidentiary basis for routine cinacalcet administration in clinical practice over time.

## Methods

We conducted a systematic review and meta-analysis of randomized controlled trials according to methods from a previously published meta-analysis, and followed a published peer-reviewed protocol [21] (Texts S1 and S2).

### Data Sources and Searches

We conducted electronic searches in the Cochrane Renal Group specialized register (through February 7, 2013) and Embase (January 1, 2012 to February 7, 2013) using search terms relevant to this review without language restriction (Table S1). The specialized register contains studies identified from the Cochrane Central Register of Controlled Trials, MEDLINE, handsearches of kidney-related journals and proceedings of major conferences, and searches of trials registries using search strategies based on the scope of the Cochrane Renal Group (http://www.cochrane-renal.org/crgtopics.php). We additionally searched Embase in the previous year (January 1, 2012, through February 7, 2013) for citations that were not automatically included in the specialized register.

### Study Selection

Two authors independently screened the search results by title and abstract, then full text, to identify potentially eligible trials that fulfilled the inclusion criteria. We considered all randomized controlled trials of any calcimimetic agent (cinacalcet HCl, NPS R-467, or NPS R-568) that reported data for adults with CKD (any stage). We defined CKD according to the National Kidney Foundation Kidney Disease Outcomes Quality Initiative, which considers CKD to be present when there are structural kidney and/or urine abnormalities with or without reduced estimated glomerular filtration rate (below 60 ml/min per 1.73 m^2^) [1]. We have used standard nomenclature, referring to having an estimated glomerular filtration rate below 60 ml/min per 1.73 m^2^ but not treated with dialysis as CKD stages 3–5, and treated with dialysis as CKD stage 5D.

### Data Extraction and Quality Assessment

Two authors independently extracted data for population characteristics, interventions, nonrandomized cointerventions, and risk of bias according to prespecified criteria from the Cochrane Collaboration's tool for assessing risk of bias [24] into a purpose-built database. Each author double-checked data extraction and data entry independently, and any discrepancies between authors were resolved by discussion. We extracted data for the following outcomes: all-cause mortality, cardiovascular mortality, parathyroidectomy, fracture, and treatment-related adverse events (including hypocalcemia, hypercalcemia, nausea, vomiting, abdominal pain, diarrhea, upper respiratory tract infection, muscle weakness or parasthesia, dyspnea, and headache). We also extracted data for end-of-treatment serum PTH, phosphorus, and calcium concentrations. Two authors independently evaluated the following risk-of-bias items using standardized methods: sequence generation, allocation concealment, blinding of patients and study personnel, blinding of outcome assessment, analysis by intention-to-treat methods, completeness of outcome data, selective reporting of outcomes, and other threats to validity (unequal treatment comparisons, early termination of trial, industry sponsor as author or involved in data handling and analysis) [24]. We also recorded whether trials published after 2005 reported trial registration in the primary trial report.

### Data Synthesis and Statistical Analysis

For dichotomous outcomes, we calculated the relative risk (RR) and 95% confidence interval (CI). For continuous outcomes, we calculated the mean difference together with a 95% CI. Where only proportions of participants experiencing an event were provided in the trial report (instead of raw event data), we estimated the number of participants experiencing one or more events by multiplying the proportion affected by the sample size, and contacted the trial authors or sponsors for additional information. We summarized effect estimates using standard and cumulative random effects meta-analysis. We assessed for heterogeneity in summary effects using the Cochran *Q* and the *I* ^2^ test (with 95% CIs) [25]. We considered a *p-*value below 0.10 to indicate significant heterogeneity. We analyzed data for all-cause mortality, cardiovascular mortality, parathyroidectomy, hypocalcemia, nausea, and vomiting within subgroups for CKD comprising adults with CKD stage 3–5 and CKD stage 5D. Insufficient data were available to determine if treatment effects differed by stage of CKD, and data were absent for kidney transplant recipients. In cumulative meta-analysis, outcome data for all-cause mortality, parathyroidectomy, hypocalcemia, and nausea from all available trials were included sequentially according to the year in which they first became available.

Additional prespecified subgroup analyses and univariate random effects metaregression were performed to explore potential sources of heterogeneity in treatment effects on all-cause mortality, parathyroidectomy, hypocalcemia, and nausea. The potential sources of heterogeneity included mean age of participants in the trial, proportion of male participants, baseline serum PTH concentration, baseline serum calcium concentration, trial duration, allocation concealment (adequate versus unclear), and year of publication. In addition, for the outcome of hypocalcemia we evaluated the serum calcium concentration used to define one or more hypocalcemia events as a source of heterogeneity in treatment effects for this outcome. Insufficient data were available to evaluate whether dialysis modality (hemodialysis versus peritoneal dialysis) modified treatment effect estimates. To assess potential bias from small-study effects, funnel plots of the log risk ratio in individual studies against the standard error of the risk ratio were generated and formally assessed for asymmetry using Egger's regression test [26]. The Duval and Tweedie trim-and-fill procedure [27] was used to quantify the possible effect of any potential publication bias evident in the meta-analyses. For all analyses, a two-tailed *p-*value<0.05 indicated statistical significance. We conducted the analyses using Comprehensive Meta-Analysis version 2 (Biostat) and SAS release 9.1 (SAS Institute).

We summarized the quality of the evidence, together with absolute treatment effects based on estimated baseline risks, using the Grading of Recommendations Assessment, Development, and Evaluation (GRADE) guidelines [28]. Definitions of evidence quality are as follows: high quality—further research is very unlikely to change our confidence in the estimate of effect; moderate quality—further research is likely to have an important impact on our confidence in the estimate of effect and may change the estimate; low quality—further research is very likely to have an important impact on our confidence in the estimate of effect and is likely to change the estimate; very low quality—any estimate of effect is very uncertain. To estimate the absolute number of people with CKD who avoided death or parathyroidectomy or incurred hypocalcemia or nausea with calcimimetic therapy, the risk estimate and 95% CI were obtained from corresponding meta-analyses, together with the absolute population risk for people with each stage of CKD, derived from cohort studies and registry data for all-cause mortality and parathyroidectomy and event rates in the control arm of available trials for hypocalcemia and nausea [5],[29],[30].

### Role of the Funding Source

This project received no specific funding. The authors had full responsibility for data collection, data interpretation, and writing of the report. The corresponding author had full access to all of the data and had the final responsibility to submit the manuscript for publication.

## Results

**Figure 1:**
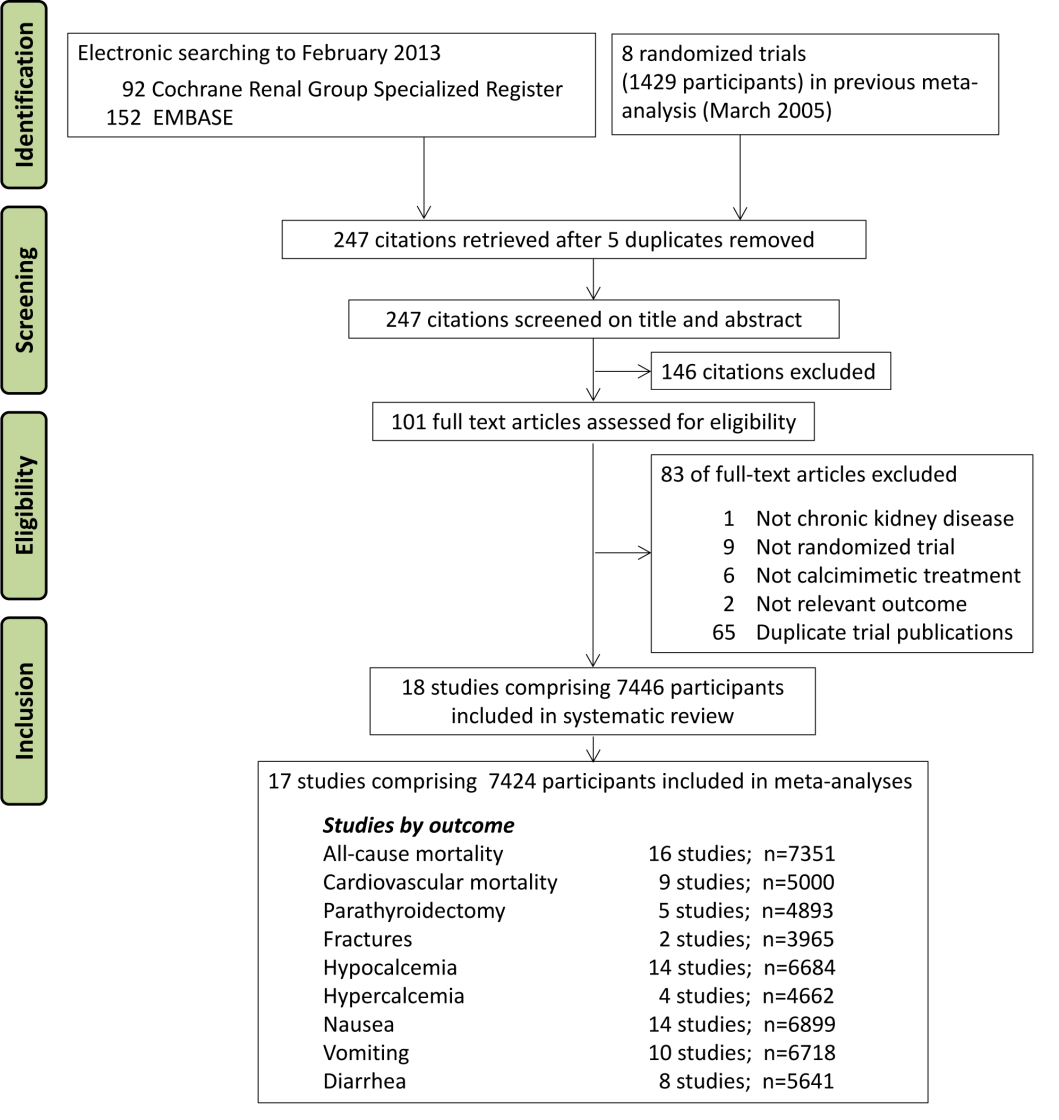
Flow chart showing number of citations retrieved by database searching, and the trials included in this review.

We included eight randomized trials comprising 1,429 participants from an earlier meta-analysis current through to March 2005 [21] and identified 244 additional citations using electronic searching (Figure 1). We included 18 trials with 7,446 adults with CKD (Table S2) in the systematic review and 17 studies with 7,424 participants in the meta-analyses. [19],[20],[23],[31]–[45].

All included trials evaluated cinacalcet hydrochloride (referred to as R-568 or AMG 073 in the four earliest trials [19],[20],[31],[32]). Cinacalcet in addition to conventional therapy (vitamin D compounds and phosphorus-binding agents) was compared against placebo and/or conventional therapy alone in all trials. In three trials, the strategy for vitamin D co-therapy differed between treatment arms [37],[43],[45]. The two earliest trials were short-term evaluations of cinacalcet treatment (8 d [20] and 15 d [19]) in adults with CKD stage 5D. Following these earliest trials of safety and biochemical efficacy, the first larger scale trial of cinacalcet therapy was reported in 2004 in 741 adults with CKD stage 5D, and measured treatment efficacy based on intact PTH concentrations [33]. Between 2004 and early 2012, 11 additional trials were reported [35]–[45], although none was primarily designed to evaluate treatment effects on patient-relevant outcomes including mortality or adverse events. In late 2012, the Evaluation of Cinacalcet Therapy to Lower Cardiovascular Events (EVOLVE) trial in 3,883 participants with CKD stage 5D was the first trial specifically designed to evaluate cinacalcet treatment on patient-centered outcomes, using a primary composite outcome of all-cause mortality or first nonfatal cardiovascular event [23].

Cinacalcet treatment was given in the included trials generally at increasing doses (usually 30 to 180 mg/d) targeted to serum PTH concentrations, with discontinuation of therapy in the event of PTH concentrations falling below a specific target and/or hypocalcemia or adverse event (described in Table S3). In one trial, the cinacalcet dose prescribed was unclear [45]. Overall, 16 trials comprised 6,988 people treated with CKD stage 5D [19],[20],[23],[31]–[34],[36]–[41],[43],[44] and two trials comprised 458 patients with CKD stages 3–5 [37],[43]. Of the 16 trials, 15 enrolled hemodialysis patients and one enrolled both hemodialysis and peritoneal dialysis patients [36]. Follow-up duration ranged between 8 d and 21.2 mo (median, 6.5 mo).

### Risk of Bias in Individual Trials

**Figure 2:**
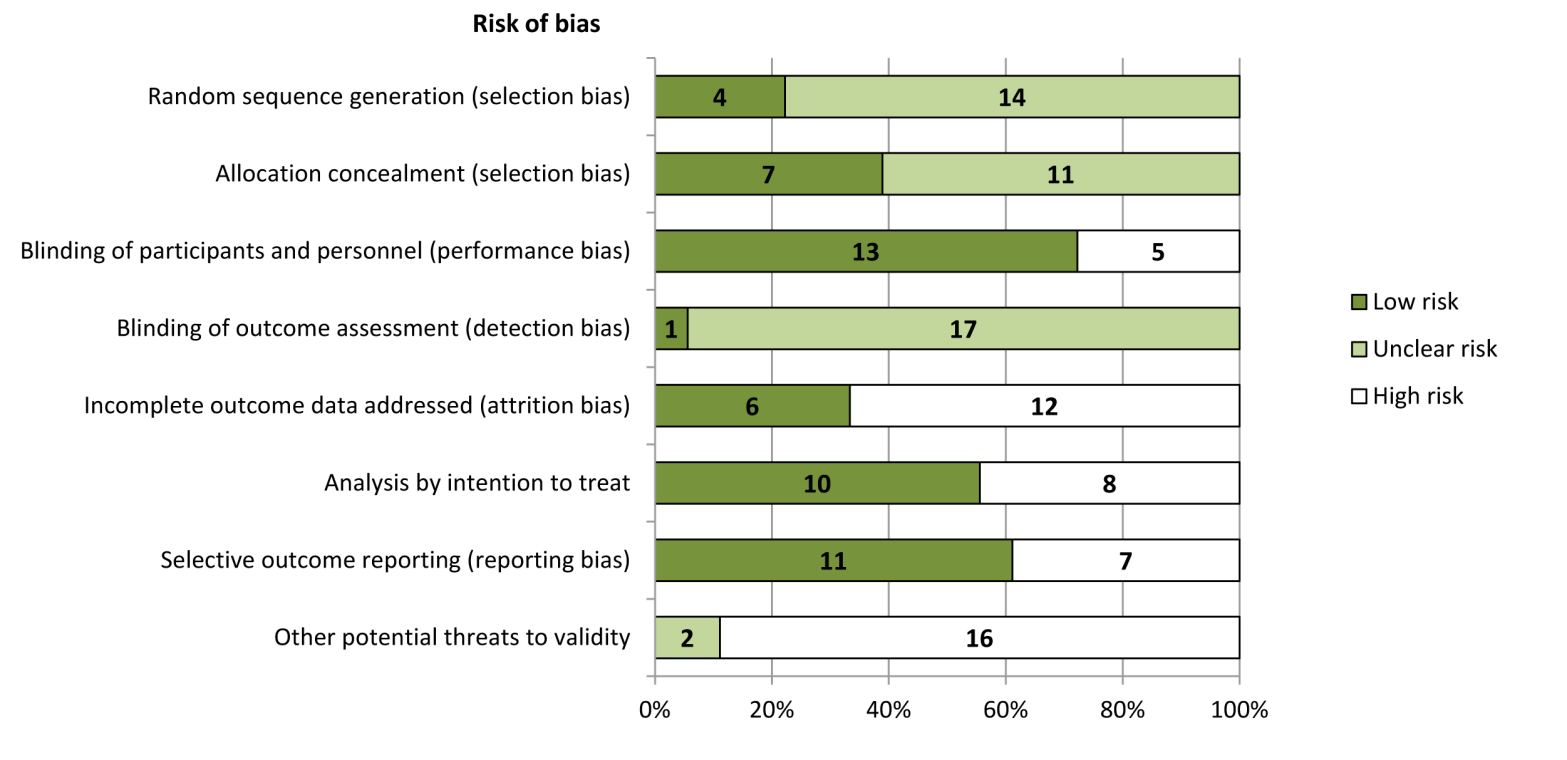
Risk of bias in included studies (***n*** **= 18).** Trials were adjudicated as free of selective reporting if they evaluated and reported the following outcomes: all-cause mortality, hypocalcemia, and two or more gastrointestinal events (nausea, vomiting, or diarrhea).

Risk of bias is summarized in Figures 2 and S1. In general, risk of bias was high or unclear in most studies for many domains we assessed. The trial sponsor had authorship and/or was involved in data collection, analysis, and interpretation in 15 trials (83%) [19],[20],[23],[31]–[37],[40]–[43],[45]. Of the ten trials reported since 2005 [23],[37]–[45], half had evidence of registration within a trials registry in the primary publication [23],[39],[42],[43],[45].

### Outcomes

#### Mortality

Compared to placebo or no treatment, cinacalcet had little or no effect on all-cause mortality (RR, 0.97 [95% CI, 0.89 to 1.05]) in high-quality evidence for people with CKD stage 5D, and imprecise effects on all-cause mortality in low-quality evidence for people with CKD stages 3–5 (RR, 0.29 [95% CI, 0.06 to 1.48]) (Figure S2; Table 1). Cinacalcet had uncertain effects on cardiovascular mortality for participants with CKD stage 5D (RR, 0.67 [95% CI, 0.16 to 2.87]) and CKD stages 3–5 (RR, 0.29 [95% CI, 0.06 to 1.48]) (Figure S3).

**Table 1:** GRADE evidence profile for effects of cinacalcet plus conventional therapy versus placebo or no treatment plus conventional therapy from meta-analyses of randomized controlled trials in people with chronic kidney disease.

Outcome^a^	Trials Reporting One or More Event Participants *n/N*	Quality Assessment					Summary of Findings				
		Study Limitations	Consistency; *I* ^2^; *p-*Value (Decrease in Quality Score)	Directness	Precision (Decrease in Quality Score)	Publication Bias (Decrease in Quality Score)	Relative Effect by Using a Random Effects Model (95% CI)	Best Estimate of Control Group Risk, Percent	Median Treatment Duration	Absolute Effect per Year of Treatment per 1,000 Treated (95% CI)^b^	Quality of Evidence^c^
**CKD stage 5D**											
All-cause mortality	9/6,502	Some limitations; sequence generation 22%; allocation concealment 44%; outcome assessment blinding 16%; ITT analysis 78%; complete follow-up 22%	No inconsistency; 0%; 0.93	Direct	No important imprecision	No important publication bias	0.97 (0.89 to 1.05)	20%	8 mo	6 fewer (22 fewer to 10 more)	High
Parathyroidectomy	5/4,893	Some limitations; sequence generation 20%; allocation concealment 40%; outcome assessment blinding 20%; ITT analysis 0%; complete follow-up 40%	No inconsistency; 0%; 0.46	Direct	No important imprecision	No important publication bias	0.49 (0.40 to 0.59)	0.7%	9 mo	3 fewer (4 fewer to 3 fewer)	High
Hypocalcemia	12/6,415	Some limitations; sequence generation 17%; allocation concealment 17%; outcome assessment blinding 8%; ITT analysis 58%; complete follow-up 33%	No inconsistency; 0%; 0.94	Direct	No important imprecision	No important publication bias	6.98 (5.10 to 9.53)	1%	7 mo	60 more (41 more to 85 more)	High
Nausea	12/6,450	Some limitations; sequence generation 25%; allocation concealment 25%; outcome assessment blinding 8%; ITT analysis 58%; complete follow-up 25%	Some inconsistency; 66%; <0.001 (−1)	Direct	No important imprecision	No important publication bias	2.02 (1.45 to 2.81)	15%	7 mo	153 more (68 more to 272 more)	Moderate
**CKD stages 3 to 5**											
All-cause mortality	2/458	Serious limitations; sequence generation 0%; allocation concealment 100%; outcome assessment blinding 0%; ITT analysis 50%; complete follow-up 50%	No inconsistency; 0%; 0.77	Direct	Imprecise (−1)	Publication bias not estimable (−1)	0.29 (0.06 to 1.48)	2.5%	6 mo	18 fewer (23 fewer to 12 more)	Low
Parathyroidectomy	0/-	Not estimable	Consistency not estimable	Directness not estimable	Imprecision not estimable	Publication bias not estimable (−1)	Not estimable	0.7%	Not estimable	Not estimable	Nil
Hypocalcemia	2/449	Serious limitations; sequence generation 0%; allocation concealment 100%; outcome assessment blinding 0%; ITT analysis 50%; complete follow-up 50%	Some inconsistency; 16%; 0.28 (−1)	Direct	Imprecise (−1)	Publication bias not estimable (−1)	31.9 (5.3 to 192.6)	1%	6 mo	310 more (43 more to 1910 more)	Very low
Nausea	2/449	Serious limitations; sequence generation 0%; allocation concealment 100%; outcome assessment blinding 0%; ITT analysis 50%; complete follow-up 50%	No important inconsistency; 6%; 0.30	Direct	No important imprecision	Publication bias not estimable (−1)	2.26 (1.29 to 3.95)	10%	6 mo	126 more (29 more to 295 more)	Low

a Data for adults treated with kidney transplantation not available.

b Approximate absolute event rates of outcomes per year are derived from previously published cohort studies and registry data for the outcomes of all-cause mortality [1],[29] and parathyroidectomy [30] or event rates in the control arm of contributing trials for outcomes of hypocalcemia and nausea. Absolute numbers of people who had CKD with mortality or parathyroidectomy events avoided or nausea or hypocalcemia events caused per 1,000 treated were calculated from the risk estimate for the outcome (and associated 95% CI) obtained from meta-analysis of placebo-controlled trials together with the absolute population risk estimates.

c Definitions of evidence quality are as follows: high quality—further research is very unlikely to change our confidence in the estimate of effect; moderate quality—further research is likely to have an important impact on our confidence in the estimate of effect and may change the estimate; low quality—further research is very likely to have an important impact on our confidence in the estimate of effect and is likely to change the estimate; very low quality—any estimate of effect is very uncertain.

ITT, intention to treat.

#### Need for parathyroidectomy

In high-quality evidence, cinacalcet prevented surgical parathyroidectomy in people with CKD stage 5D (RR, 0.49 [95% CI, 0.40 to 0.59]) (Figure S4). Data for treatment effects on parathyroidectomy were not available for adults with CKD stages 3–5.

#### Fracture

In two trials in adults with CKD stage 5D, cinacalcet had imprecise and inconsistent effects on fracture (RR, 0.52 [95% CI, 0.12 to 2.27]) (Figure S5), and data were not available for adults with CKD stages 3–5.

#### Adverse events

Definitions of hypocalcemia and hypercalcemia in included trials are provided in Table S3. The cutoff for the definition of hypocalcemia generally ranged between 1.88 and 2.10 mmol/l and that of hypercalcemia was 2.55 or 2.63 mg/dl. In all stages of CKD, cinacalcet increased the risk of hypocalcemia (RR, 7.38 [95% CI, 5.43 to 10.03]) (Figure S6), nausea (RR, 2.05 [95% CI, 1.54 to 2.75]), vomiting (RR, 1.95 [95% CI, 1.74 to 2.18]) (Figure S7), and diarrhea (RR, 1.15 [95% CI, 1.02 to 1.29]) (Table 2). Cinacalcet therapy reduced the risk of hypercalcemia (RR, 0.23 [95% CI, 0.05 to 0.97]) (Figure S8). Cinacalcet had uncertain effects on abdominal pain, upper respiratory tract infection, muscle weakness or parasthesia, dyspnea, and headache (Table 2).

**Table 2:** Summary of adverse effects for cinacalcet plus conventional therapy versus placebo or no treatment plus conventional therapy for adults with chronic kidney disease (any stage).

Outcome	Number of Studies	Number of Participants	RR (Random Effects)	*I* ^2^ (Percent) [95% CI]	*p-*Value for Heterogeneity
Hypocalcemia	14	6,864	7.38 (5.43 to 10.03)	0	0.73
Hypercalcemia	4	4,662	0.23 (0.05 to 0.97)	77 [62–86]	0.005
Nausea	14	6,899	2.05 (1.54 to 2.75)	62 [49–71]	0.001
Vomiting	10	6,718	1.95 (1.74 to 2.18)	0	0.50
Diarrhea	8	5,641	1.15 (1.02 to 1.29)	0	0.61
Abdominal pain	4	831	1.62 (0.55 to 4.82)	70 [49–82]	0.02
Upper respiratory tract infection	4	1,856	0.95 (0.39 to 2.33)	80 [67–87]	0.002
Muscle weakness or parasthesia	3	469	1.99 (0.70 to 5.67)	0	0.88
Dyspnea	2	250	1.02 (0.49 to 2.12)	0	0.38
Headache	3	1,115	1.51 (0.95 to 2.42)	0	0.69

#### Serum parathyroid hormone, phosphorus, and calcium concentrations

Cinacalcet decreased serum PTH (mean difference, −281 ng/l [95% CI, −326 to −236]) and calcium concentrations (mean difference, −0.22 mmol/l [95% CI, −0.25 to −0.19]), but had little or no effect on serum phosphorus concentrations (mean difference, −0.07 mmol/l [95% CI, −0.19 to 0.04]) (Table S4).

### Cumulative Meta-Analysis

**Figure 3:**
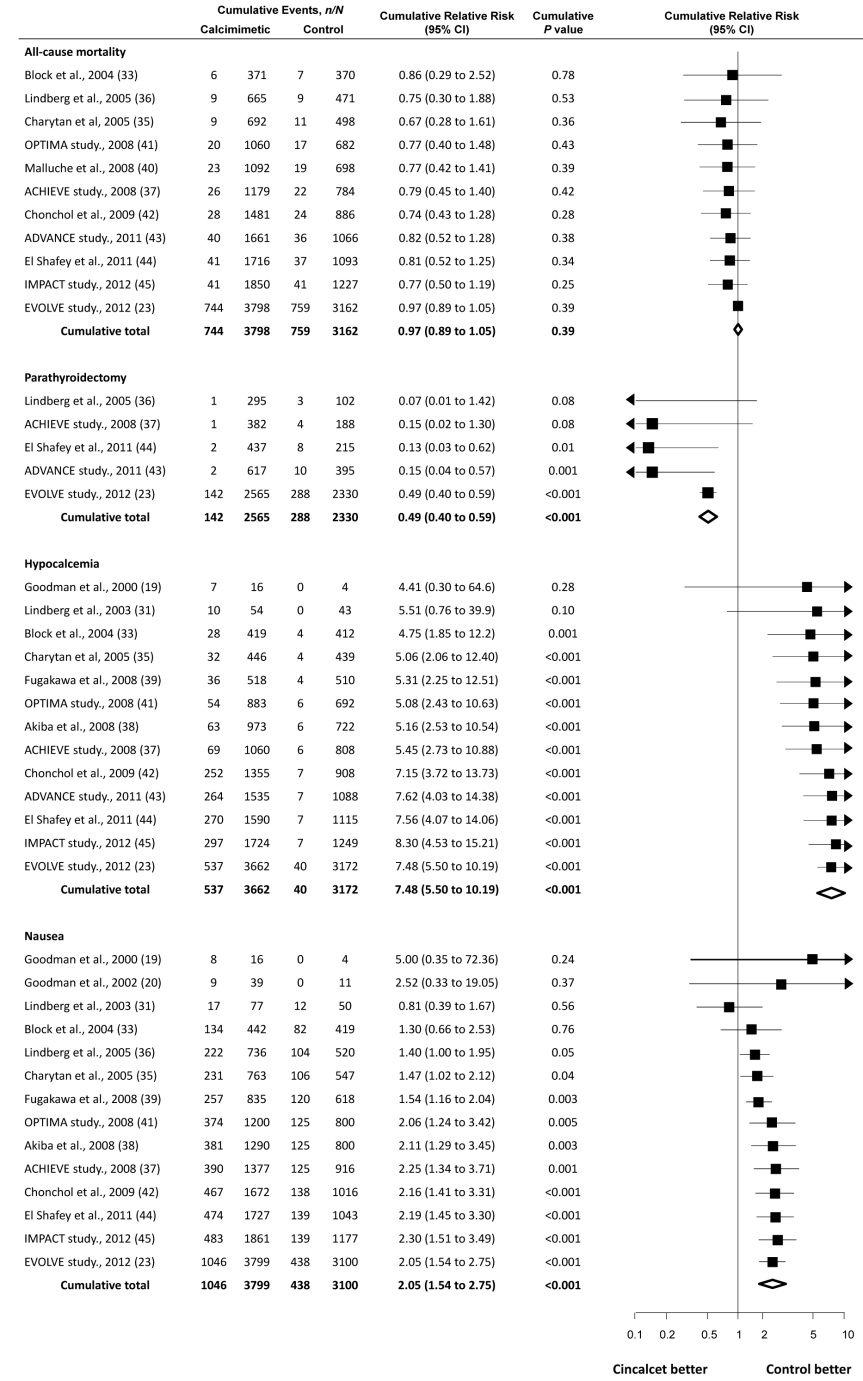
Cumulative meta-analysis of randomized trials comparing cinacalcet plus conventional therapy versus placebo or no treatment plus conventional therapy. Studies are listed by first author or study name, year, and reference number (in parentheses).

Although the first large randomized trial designed to evaluate effects of cinacalcet on mortality and cardiovascular events reported its results only recently [23], cumulative meta-analysis shows that it and numerous smaller trials conducted since 2000 have collectively signaled limited benefit for mortality, and increased hypocalcemia and nausea (Figure 3). Specifically for mortality, although the largest trial (EVOLVE) [23], including nearly 4,000 participants, has just reported and increased our certainty that cinacalcet therapy has little or no effect on risks of death, cumulative treatment estimates for cinacalcet have not changed materially over the previous decade. Similarly, when examined collectively, earlier trials had identified the markedly increased risks of hypocalcemia and nausea by 2005. Since then, the accrual of approximately 6,000 further participants in additional trials has not substantively altered estimates of excess side effects, although they have increased our certainty in treatment-related adverse outcomes. Notably for parathyroidectomy, the EVOLVE trial [23] has increased our confidence in the effect of cinacalcet treatment and indicates smaller treatment benefits than were observed in earlier studies.

### Exploration of Heterogeneity and Sensitivity Analyses

Using univariate metaregression and subgroup analyses, we explored mean age of participants in the trial, gender, baseline serum PTH and calcium concentrations, duration of follow-up, allocation concealment, and year of publication as sources of heterogeneity in treatment effects for all-cause mortality, parathyroidectomy, hypocalcemia, and nausea. Analyses showed that none of these variables modified estimates for all-cause mortality or parathyroidectomy. Increasing trial-level age was associated with increased risk of hypocalcemia and nausea (Table S5). We also evaluated the serum calcium concentration used to define hypocalcemia in individual trials as a source of heterogeneity and observed that, as might be predicted, use of a higher cut point for serum calcium to define hypocalcemia was associated with an increased risk of this outcome. In subgroup analyses, trials in which allocation concealment was adequate had statistically similar estimates of treatment effect (mortality RR, 0.97 [95% CI, 0.89 to 1.05]; hypocalcemia RR, 8.00 [95% CI, 5.66 to 11.32]; nausea RR, 2.03 [95% CI, 1.21 to 3.40]) to those trials that had unclear allocation concealment (mortality RR, 0.92 [95% CI, 0.56 to 1.51]; hypocalcemia RR, 5.84 [95% CI, 2.98 to 11.43]; nausea RR, 2.31 [95% CI, 1.42 to 3.77]) (*p*-value for subgroup differences >0.4 for all). Subgroup analyses for effects of allocation concealment were not possible for parathyroidectomy.

When we excluded the three trials in which randomized cointervention strategies for vitamin D compounds were not comparable between treatment arms [37],[43],[45], we observed similar treatment estimates in dialysis patients (all-cause mortality RR, 0.97 [95% CI, 0.89 to 1.05]; cardiovascular mortality RR, 0.95 [95% CI, 0.84 to 1.08]; hypocalcemia RR, 6.72 [95% CI, 4.88 to 9.25]; nausea RR, 1.89 [95% CI, 1.38 to 2.60]; vomiting RR, 1.98 [95% CI, 1.71 to 2.30]), although risks of hypercalcemia became less certain (RR, 0.88 [95% CI, 0.55 to 1.41]). When we limited the analysis of all-cause mortality to trials that had a follow-up of 6 mo or longer, we found the treatment effect was unchanged (RR, 0.97 [95% CI, 0.90 to 1.05]). We observed asymmetry in the funnel plot for the outcome of all-cause mortality, suggesting that small studies remained unpublished (Egger's regression test, *p* = 0.01). When we imputed five potentially missing studies, the risk of all-cause mortality remained unchanged (RR, 0.97 [95% CI, 0.89 to 1.05]) (Figure S9). No asymmetry was observed in funnel plots for hypocalcemia or nausea, and data for parathyroidectomy were insufficient to allow for detection of small-study effects.

We conducted sensitivity analyses to check the robustness of treatment effect estimates and their precision when trials in which zero events had occurred in one or both arms were available. We used a continuity correction of 0.5 added to all cells for such trials and found no substantive difference in the results. In addition to the cumulative meta-analysis reporting treatment estimates over time for all-cause mortality, parathyroidectomy, hypocalcemia, and nausea, we evaluated the cumulative treatment effect estimates for cardiovascular mortality before and after the inclusion of the large EVOLVE trial [23]. In the absence of EVOLVE, the summary RR for cardiovascular mortality was 0.25 (95% CI, 0.06 to 1.02), while including EVOLVE provided a RR of 0.68 (95% CI, 0.32 to 1.45).

## Discussion

In high- to moderate-quality evidence from 16 randomized controlled trials involving 6,988 patients, routine cinacalcet (30 to 180 mg/d) therapy in people with CKD stage 5D decreases PTH concentrations (281 ng/l [32 pmol/l]), reduces hypercalcemia, and infrequently prevents surgical parathyroidectomy, but has little or no effect on all-cause mortality, has imprecise effects on cardiovascular death, and is associated commonly with adverse effects including hypocalcemia, nausea, vomiting, and diarrhea. On average, routinely treating 1,000 people for 1 y has no effect on mortality, might prevent three patients from experiencing surgical parathyroidectomy, and leads to approximately 60 and 150 patients experiencing hypocalcemia and nausea, respectively. Evidence in people with CKD stages 3–5 is scant and generally low or very low quality. Because of lower absolute risks of parathyroidectomy in earlier stages of CKD, the benefits of cinacalcet identified in dialysis populations are likely to be smaller if generalized to people with CKD stages 3–5. Data for recipients of a kidney transplant and those treated with peritoneal dialysis were largely absent.

Although it remains possible that routine cinacalcet prescribing has a beneficial effect on all-cause mortality, consistent treatment effects across all the available studies providing data suggest that, at best, any benefit for mortality is likely to be small. Given that lag censoring analyses for outcomes (where data were censored 6 mo after patients stopped using the study drug) were reported as prespecified secondary analyses in the EVOLVE trial [23] and suggested a potential benefit for cinacalcet on total mortality (hazard ratio, 0.83 [95% CI, 0.73 to 0.96]), it might be argued that additional trials of cinacalcet are now needed or that cinacalcet lowers mortality. However, we suggest that, given that lag censoring approaches were secondary analyses and that overall data for mortality in this meta-analysis are high-quality according to GRADE criteria, additional placebo-controlled trials of cinacalcet are very unlikely to change the confidence in the size and direction of the treatment estimates we observed. By contrast, given the low- to very-low-quality evidence currently available for people with CKD stages 3–5, and the lack of available data to allow analysis of whether treatment effects differ by stage of CKD, additional trial data for this specific group of patients would be informative.

Notably, the trials contributing to the analyses all sought to investigate the use of cinacalcet as “routine” or “first line” therapy for elevated PTH levels. Their findings therefore do not exclude the possibility that cinacalcet may afford benefits in the treatment of elevated PTH levels resistant to treatment with vitamin D compounds and phosphate binders. The generally negative findings of existing trials on patient-level end points have resulted in clinical practice guideline recommendations that suggest that cinacalcet should be used when serum parathyroid levels are very high, other treatments have been ineffective, and surgical parathyroidectomy is contraindicated [46]. However, the specific use of cinacalcet in this clinical setting has not been adequately evaluated in randomized trials, and, in particular, outcomes and adverse events after parathyroidectomy versus cinacalcet have not been studied.

Before the development of cinacalcet, vitamin D compounds were the mainstay of therapy to normalize perturbed PTH concentrations in CKD, which if left unchecked lead to painful fractures, bone deformity, and generalized osteopenia. In a now familiar sequence of events in nephrology, although vitamin D therapy was effective for improving a surrogate outcome (lower serum PTH levels) and was associated with lower mortality in nonrandomized studies [47], subsequent randomized trials did not clearly demonstrate beneficial effects of vitamin D compounds on cardiovascular events or death for people with CKD [13]. Similarly, while cinacalcet was shown 10 y ago to markedly improve surrogate outcomes (both serum PTH and calcium by phosphorus product levels) in people with CKD [20], and observational analyses suggest an association between cinacalcet treatment and improved all-cause and cardiovascular mortality [17], until recently, randomized trial evidence systematically evaluating the effect of cinacalcet on clinical outcomes was not available. Despite this vacuum of high-quality evidence for patient-centered end points and cumulative data indicating frequent side effects including hypocalcemia, nausea, or vomiting, cinacalcet has become the most expensive drug cost and the eighth most frequently prescribed drug for Medicare Part D enrolled dialysis patients in the United States, and year-on-year prescribing costs are increasing rapidly in the United Kingdom [5],[22].

This meta-analysis shows that, although its use is widespread and costly, cinacalcet provides small absolute benefits for parathyroidectomy, provides no reductions in mortality, and frequently leads to adverse gastrointestinal effects that may adversely influence nutrition and quality of life in these patients. Importantly, the effects of cinacalcet treatment on all-cause mortality, parathyroidectomy, hypocalcemia, and nausea were all identifiable before the EVOLVE trial [23] was released in late 2012, and the EVOLVE trial has now largely only increased our confidence in treatment effects. The EVOLVE trial has additionally provided us with important data for cardiovascular mortality, showing that benefits of therapy on this outcome are lower than cumulatively estimated by earlier trials. The EVOLVE trial was needed to provide certainty and high-quality data that routine cinacalcet use provides little or no benefit for adults treated with dialysis; monitoring of prescribing data post-EVOLVE may now reveal a fall in the prescribing costs and frequency of routine cinacalcet administration in parallel with the high-quality evidence available, although questions will remain as to why prescribing costs became so high in the context of insufficient cumulated evidence over the last decade. As with vitamin D compounds previously, the pathway from drug development to clinical use for cinacalcet reminds us that relying on surrogate end points and nonrandomized studies to evaluate treatment efficacy for new interventions is likely to result in unreliable estimates of clinical effectiveness. This, in turn, leads to extensive use of interventions that do not improve population outcomes and unnecessarily increase healthcare expenditure.

The treatment effect we observed for cinacalcet on fracture (RR, 0.53) was similar in magnitude to, but less certain than, the risk estimate observed in a pooled analysis of four similarly designed randomized, double-blind, placebo-controlled trials of cinacalcet enrolling 1,184 participants with CKD stage 5D and intact PTH concentrations of 300 ng/l or more, in which the RR of fracture was 0.46 (95% CI, 0.22–0.95) [18]. It was unclear in that publication which trials were included in the pooled analysis, from which data for extended treatment in two trials including about half the randomized participants were included. It is possible that cinacalcet lowers the risk of fracture, but at this time, treatment estimates based on published trial data summarized by meta-analysis are imprecise and lower quality.

The current evidence for cinacalcet in this systematic review is consistent with the UK National Health Service National Institute for Health and Clinical Excellence guidance recommending that cinacalcet should not be used for the routine treatment of elevated serum PTH levels in people with CKD and should be limited to people with elevated PTH concentrations refractory to standard therapy, with a normal or high serum calcium concentration, *and* in whom surgical parathyroidectomy is contraindicated because the risks of surgery outweigh the benefits [46]. The data also support the current US Food and Drug Administration approval for cinacalcet, which is restricted to patients with CKD stage 5D who have secondary hyperparathyroidism, although benefits of treatment in this setting are limited to prevention of surgical parathyroidectomy and avoidance of hypercalcemia [14]. At this time, however, the available randomized evidence for cinacalcet does not support the current Kidney Disease: Improving Global Outcomes clinical practice guidelines suggesting that people with CKD treated with dialysis and elevated or rising PTH levels (beyond two to nine times the upper normal limit) receive vitamin D compounds or calcimimetics or a combination to decrease serum PTH levels to within the suggested range [11].

Although based on a peer-reviewed protocol and conducted using methods developed by the Cochrane Collaboration, our review has limitations that should be considered. First, data for cinacalcet therapy were largely limited to adults with CKD stage 5D. Insufficient data were available to determine whether treatment effects differed according to severity of CKD. Second, data for recipients of a kidney transplant were absent, although as in other stages of CKD, cinacalcet use may provide benefits outweighing treatment hazards for people requiring parathyroidectomy in whom surgical therapy is contraindicated. Third, due to a relative absence of trials in patients receiving peritoneal dialysis, treatment estimates for this specific group are uncertain. Finally, because of the lack of head-to-head data in available trials, the comparative effectiveness of cinacalcet versus vitamin D compounds for patient-level outcomes remains uncertain.

In conclusion, cinacalcet therapy provides small reductions in the risk of surgical parathyroidectomy but has little or no effect on all-cause mortality and uncertain effects on cardiovascular death for people with CKD and is commonly associated with nausea and vomiting. Routine use of cinacalcet therapy in people with CKD does not appear warranted, and benefits may be limited to preventing parathyroidectomy in the small number of patients for whom surgery is contraindicated. Additional trials in patients with CKD stage 5D are unlikely to change the estimates of treatment effects for cinacalcet.

## Supporting Information

Figure S1 **Risk of bias in trials of cinacalcet therapy versus conventional treatment in adults with chronic kidney disease.**
(TIF)

Figure S2 **Effect of cinacalcet plus conventional therapy versus placebo or no treatment plus conventional therapy on all-cause mortality in adults with chronic kidney disease.**
(TIF)

Figure S3 **Effect of cinacalcet plus conventional therapy versus placebo or no treatment plus conventional therapy on cardiovascular mortality in adults with chronic kidney disease treated with dialysis.**
(TIF)

Figure S4 **Effect of cinacalcet plus conventional therapy versus placebo or no treatment plus conventional therapy on parathyroidectomy in adults with chronic kidney disease treated with dialysis.**
(TIF)

Figure S5 **Effect of cinacalcet plus conventional therapy versus placebo or no treatment plus conventional therapy on fracture in adults with chronic kidney disease treated with dialysis.**
(TIF)

Figure S6 **Effect of cinacalcet plus conventional therapy versus placebo or no treatment plus conventional therapy on hypocalcemia in adults with chronic kidney disease.**
(TIF)

Figure S7 **Effect of cinacalcet plus conventional therapy versus placebo or no treatment plus conventional therapy on nausea and vomiting in adults with chronic kidney disease.**
(TIF)

Figure S8 **Effect of cinacalcet plus conventional therapy versus placebo or no treatment plus conventional therapy on hypercalcemia in adults with chronic kidney disease.**
(TIF)

Figure S9 **Funnel plot to assess bias in estimates of all-cause mortality caused by small-study effects.** Funnel plot assessing for potential publication bias. Individual studies reporting one or more events (*n* = 11), together with a diamond denoting the log rate ratio and 95% CI for actual studies, are shown in blue. Imputed hypothetical studies (*n* = 5) inserted using the Duval and Tweedie trim-and-fill method to account for missing studies with a lower risk for all-cause mortality are shown, together with the associated log rate ratio and its 95% CI, in red. The risk estimate for all-cause mortality adjusted for potentially missing studies is 0.97 (95% CI, 0.90 to 1.05).
(TIF)

Table S1 **Electronic search strategies.**
(PDF)

Table S2 **Included studies comparing cinacalcet plus conventional therapy versus placebo or no treatment plus conventional therapy in adults with chronic kidney disease.**
(PDF)

Table S3 **Definitions of parathyroid hormone and calcium targets triggering reduction in cinacalcet dose, and definition of hypocalcemia and hypercalcemia end points in included trials.**
(PDF)

Table S4 **Effects of cinacalcet plus conventional therapy versus placebo or no treatment plus conventional therapy on end-of-treatment serum parathyroid hormone, phosphorus, and calcium concentrations in adults with chronic kidney disease.**
(PDF)

Table S5 **Univariate metaregression exploring the role of patient and trial characteristics in the effect of cinacalcet therapy on clinical outcomes.**
(PDF)

Text S1 **PRISMA checklist.**
(DOC)

Text S2 **Protocol.**
(PDF)

## Abbreviations

CI: confidence interval
CKD: chronic kidney disease
EVOLVE: Evaluation of Cinacalcet Therapy to Lower Cardiovascular Events
GRADE: Grading of Recommendations Assessment, Development, and Evaluation
PTH: parathyroid hormone
RR: relative risk
