# Supplementary figures and images for: Cinacalcet in Patients with Chronic Kidney Disease: A Cumulative Meta-Analysis of Randomized Controlled Trials
Source: PLoS Med. 2013 Apr 30;10(4):e1001436. doi: 10.1371/journal.pmed.1001436 (PMC3640084; doi:10.1371/journal.pmed.1001436)

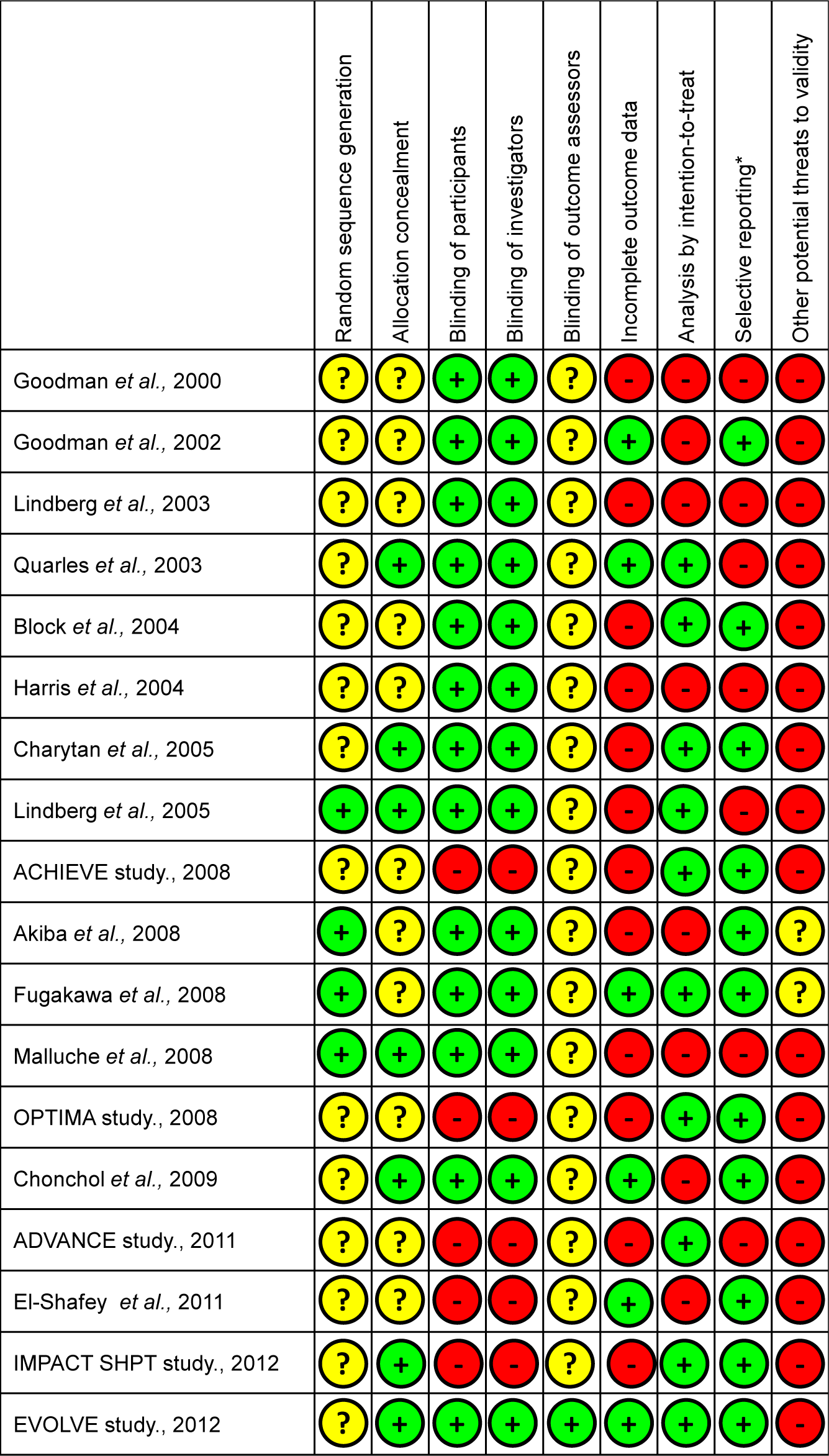

Supplement: Figure S1 — Risk of bias in trials of cinacalcet therapy versus conventional treatment in adults with chronic kidney disease. (TIF) [file pmed.1001436.s001.tif]

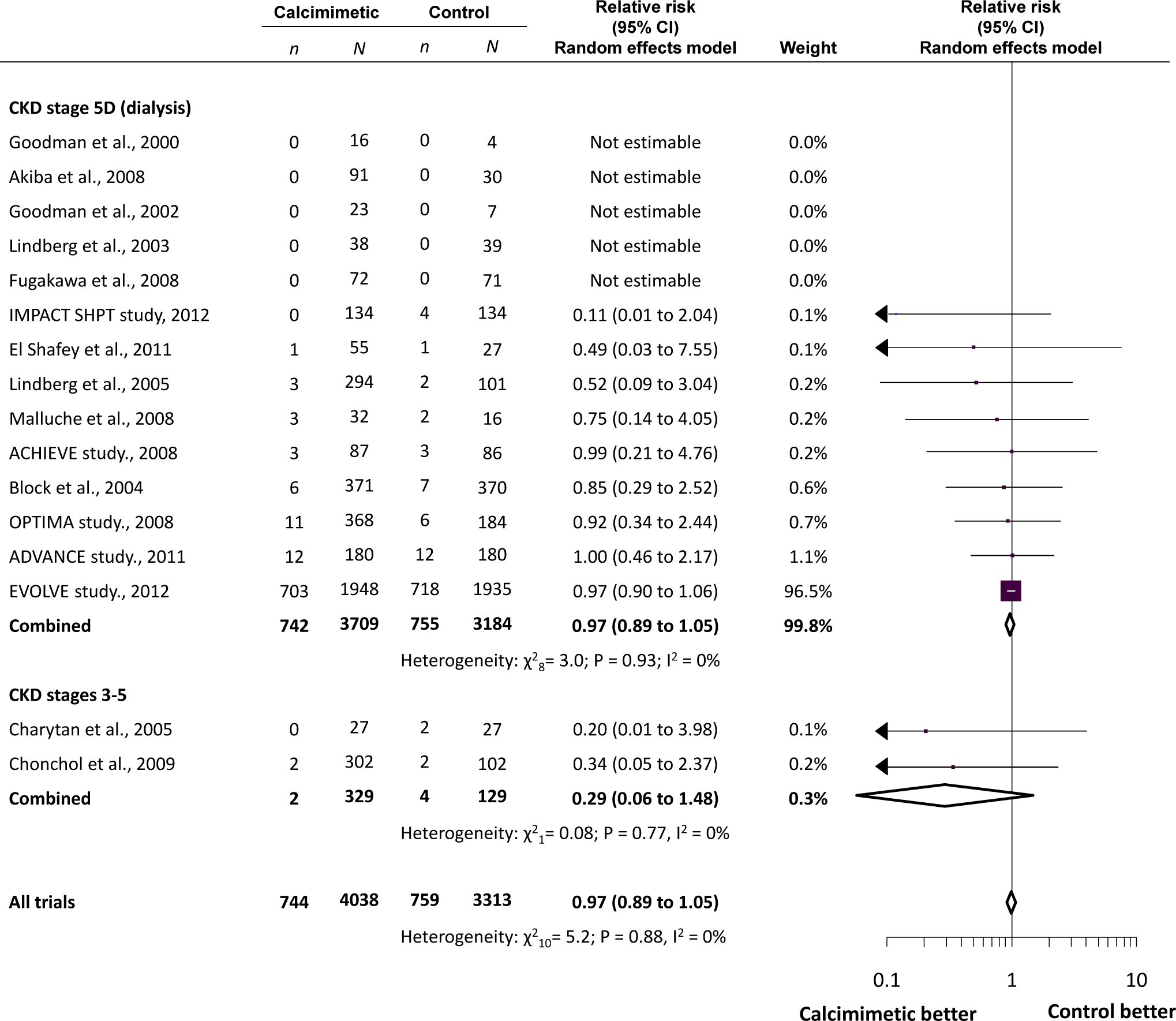

Supplement: Figure S2 — Effect of cinacalcet plus conventional therapy versus placebo or no treatment plus conventional therapy on all-cause mortality in adults with chronic kidney disease. (TIF) [file pmed.1001436.s002.tif]

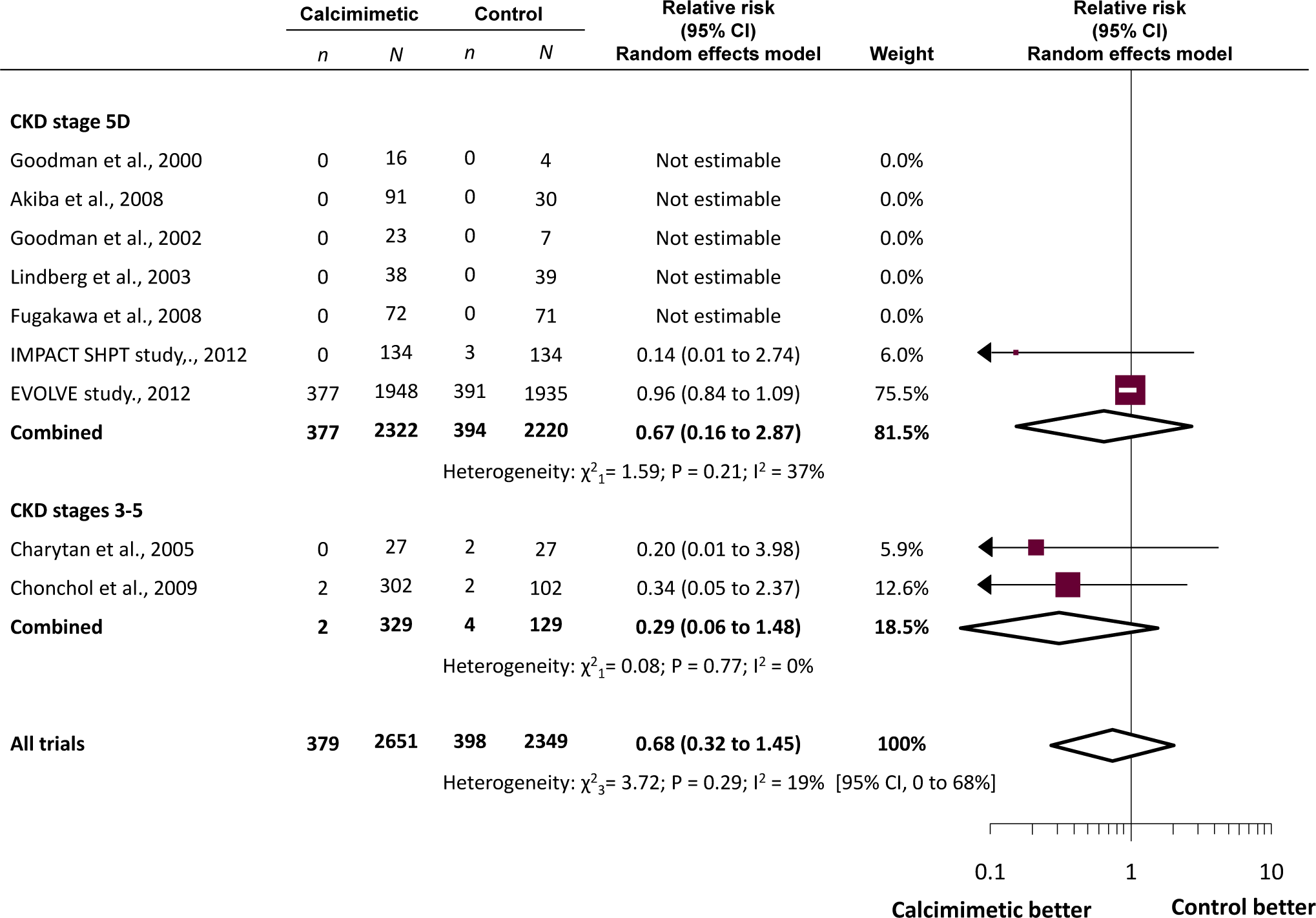

Supplement: Figure S3 — Effect of cinacalcet plus conventional therapy versus placebo or no treatment plus conventional therapy on cardiovascular mortality in adults with chronic kidney disease treated with dialysis. (TIF) [file pmed.1001436.s003.tif]

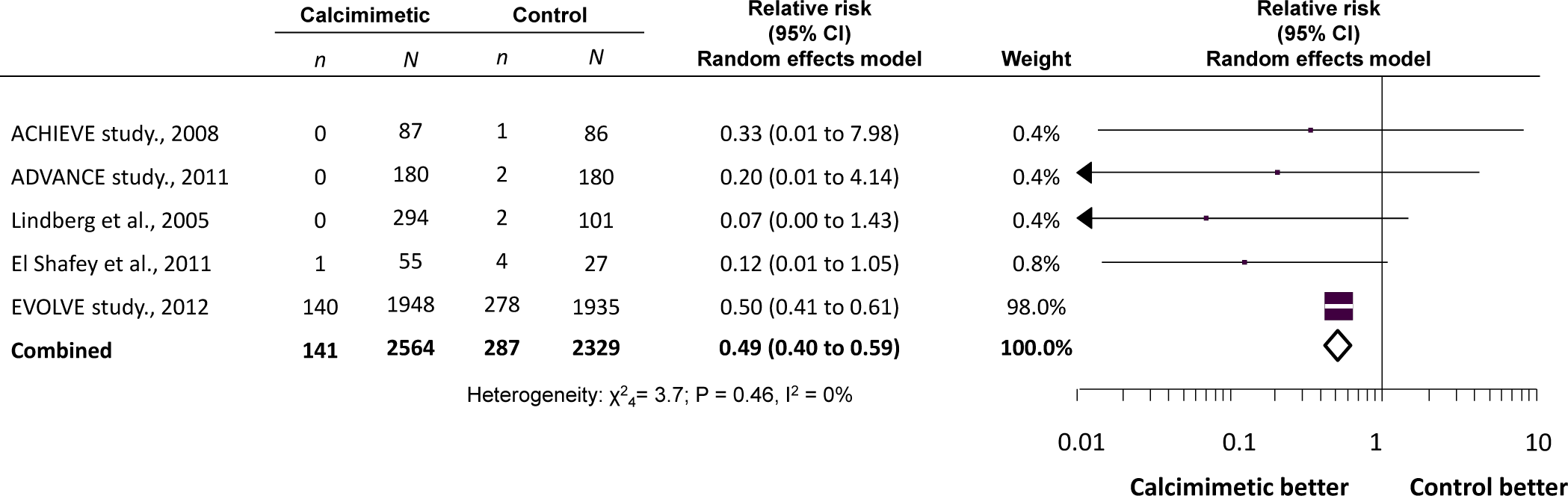

Supplement: Figure S4 — Effect of cinacalcet plus conventional therapy versus placebo or no treatment plus conventional therapy on parathyroidectomy in adults with chronic kidney disease treated with dialysis. (TIF) [file pmed.1001436.s004.tif]

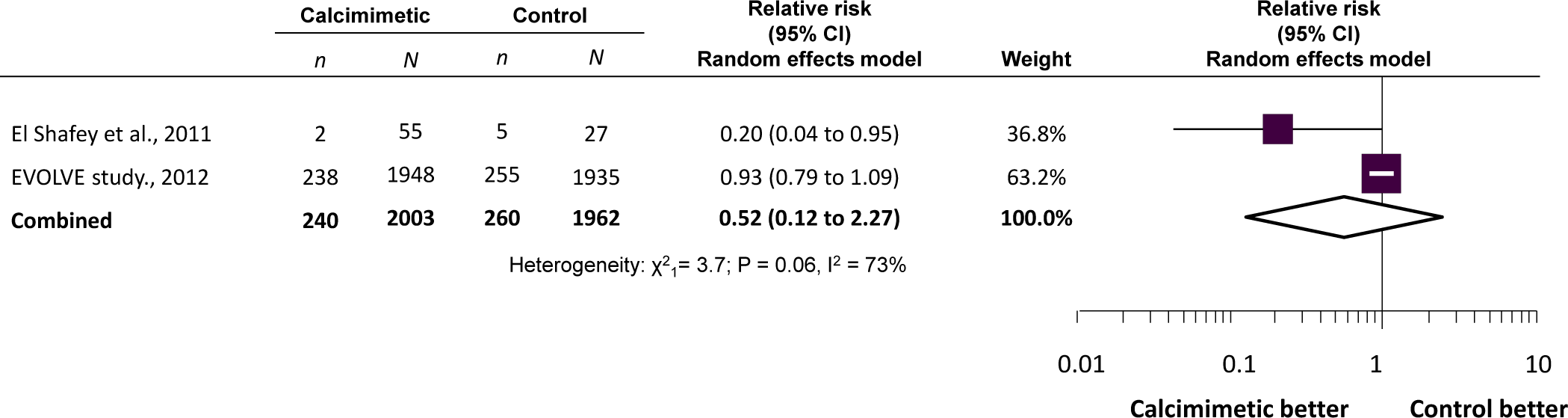

Supplement: Figure S5 — Effect of cinacalcet plus conventional therapy versus placebo or no treatment plus conventional therapy on fracture in adults with chronic kidney disease treated with dialysis. (TIF) [file pmed.1001436.s005.tif]

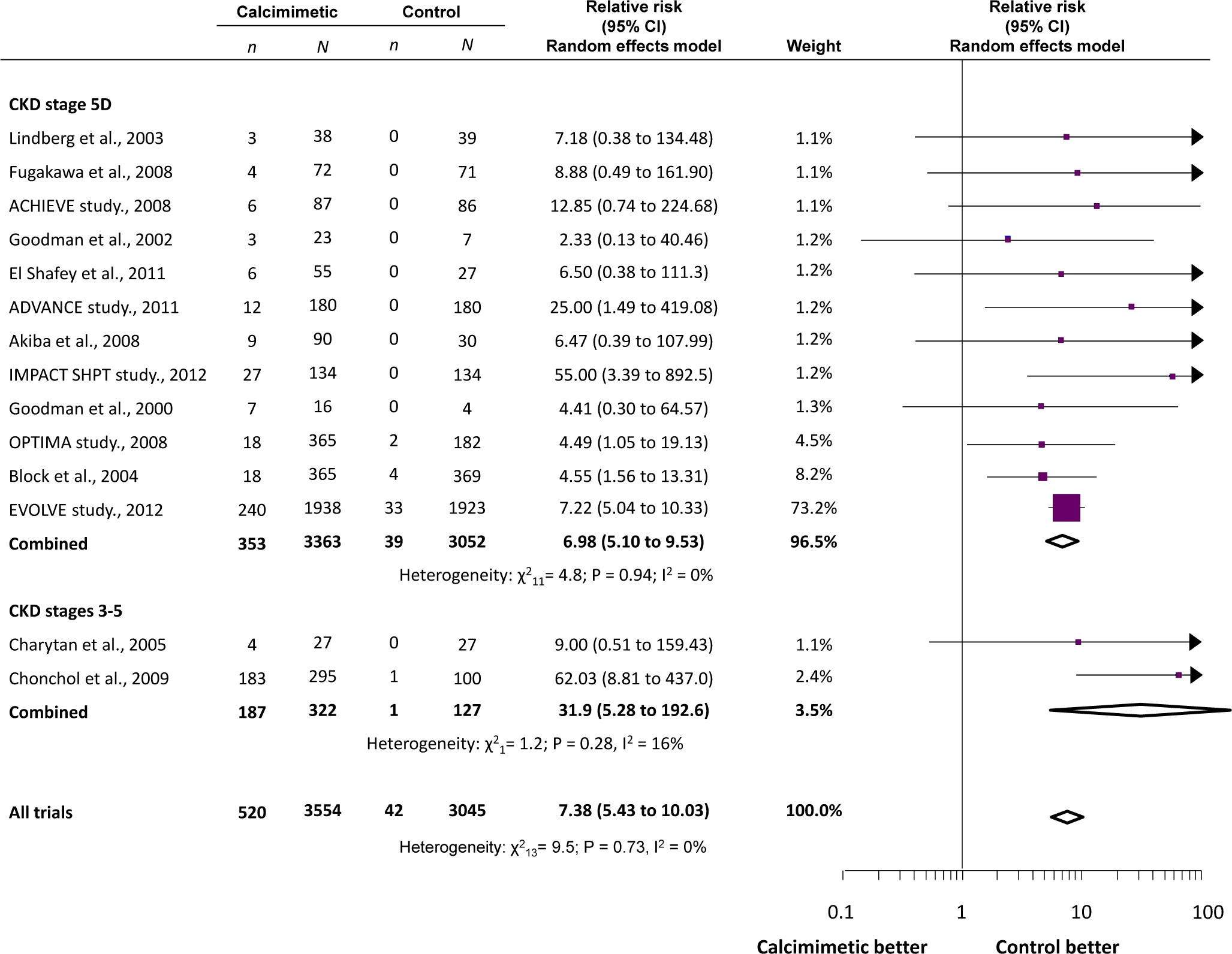

Supplement: Figure S6 — Effect of cinacalcet plus conventional therapy versus placebo or no treatment plus conventional therapy on hypocalcemia in adults with chronic kidney disease. (TIF) [file pmed.1001436.s006.tif]

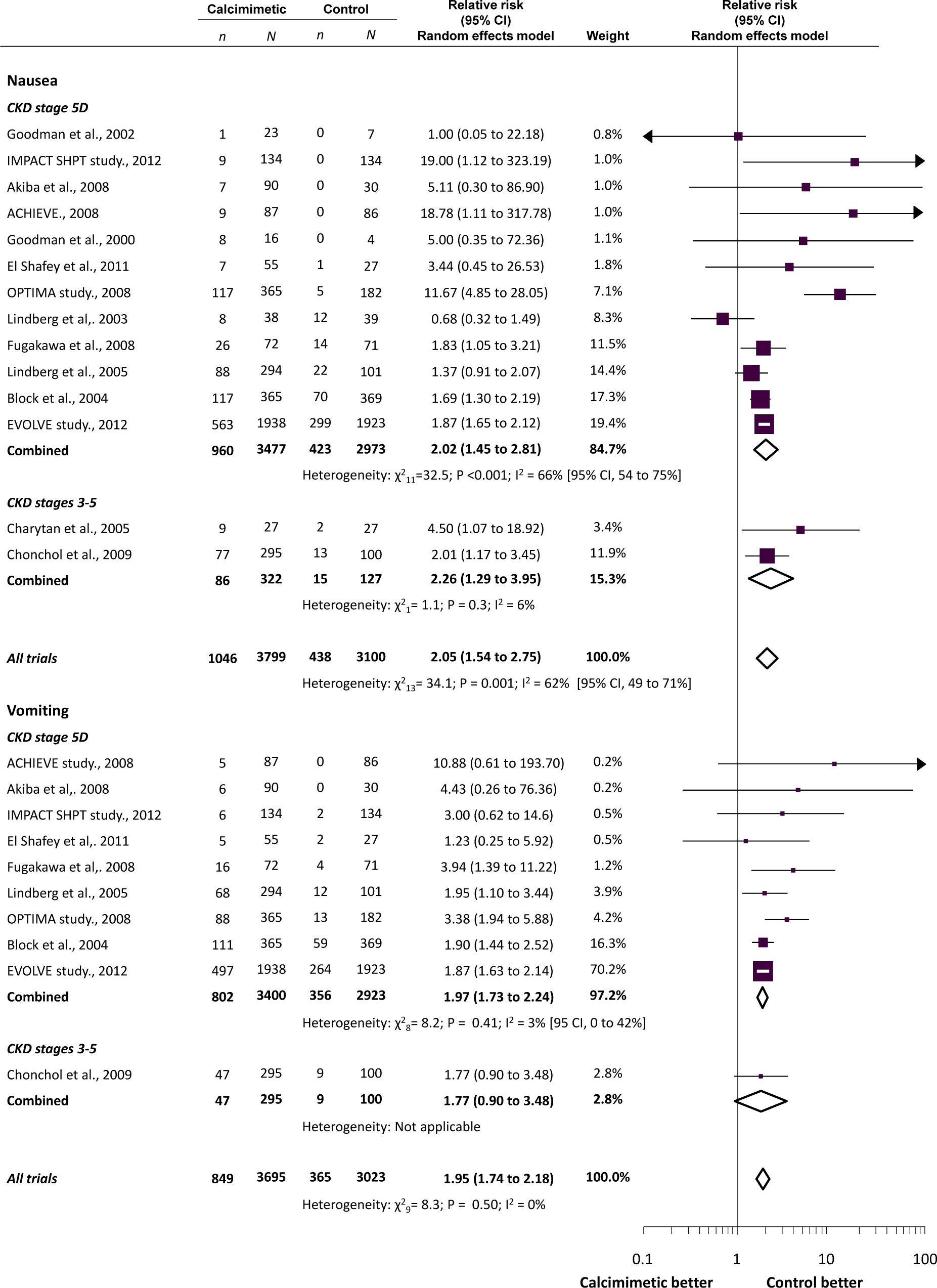

Supplement: Figure S7 — Effect of cinacalcet plus conventional therapy versus placebo or no treatment plus conventional therapy on nausea and vomiting in adults with chronic kidney disease. (TIF) [file pmed.1001436.s007.tif]

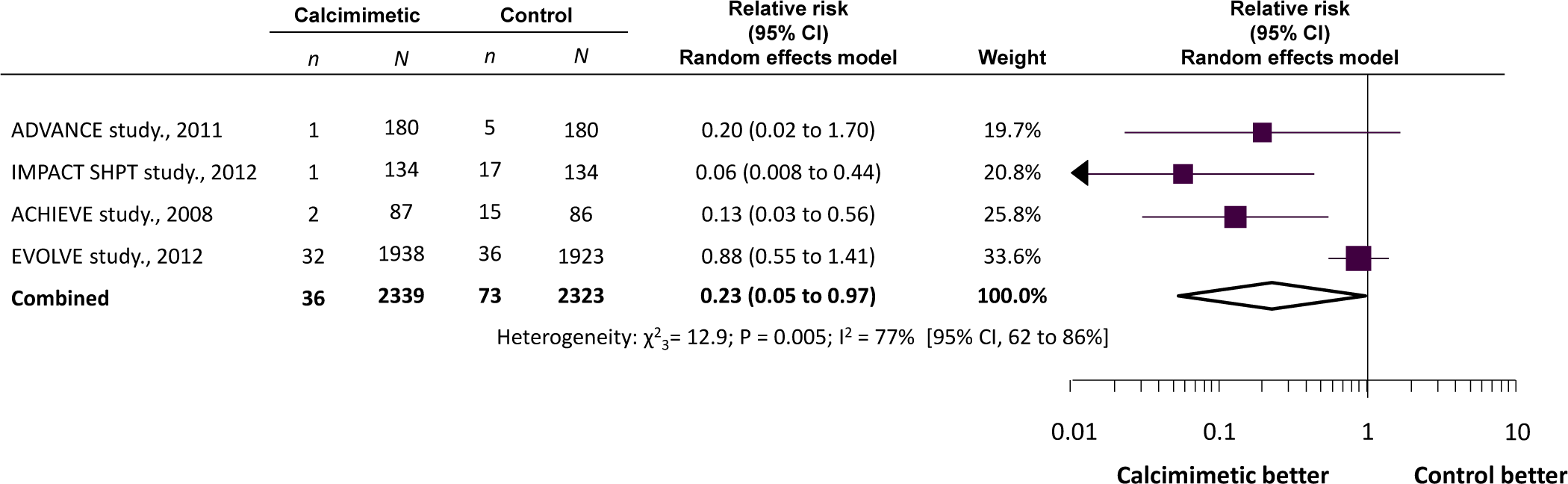

Supplement: Figure S8 — Effect of cinacalcet plus conventional therapy versus placebo or no treatment plus conventional therapy on hypercalcemia in adults with chronic kidney disease. (TIF) [file pmed.1001436.s008.tif]

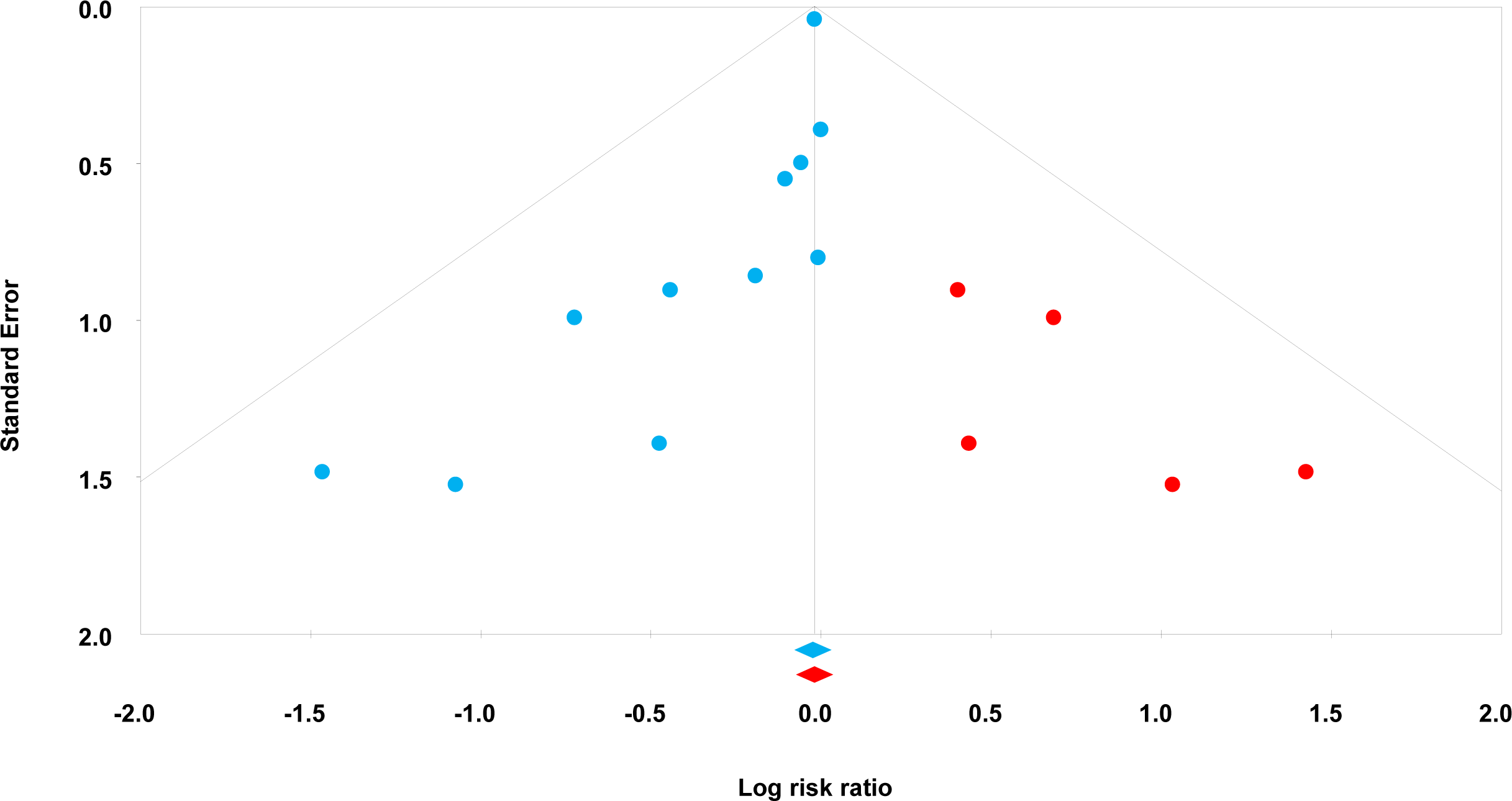

Supplement: Figure S9 — Funnel plot to assess bias in estimates of all-cause mortality caused by small-study effects. Funnel plot assessing for potential publication bias. Individual studies reporting one or more events (n = 11), together with a diamond denoting the log rate ratio and 95% CI for actual studies, are shown in blue. Imputed hypothetical studies (n = 5) inserted using the Duval and Tweedie trim-and-fill method to account for missing studies with a lower risk for all-cause mortality are shown, together with the associated log rate ratio and its 95% CI, in red. The risk estimate for all-cause mortality adjusted for potentially missing studies is 0.97 (95% CI, 0.90 to 1.05). (TIF) [file pmed.1001436.s009.tif]
