# Supplementary material for: Cinacalcet in Patients with Chronic Kidney Disease: A Cumulative Meta-Analysis of Randomized Controlled Trials
Source: PLoS Med. 2013 Apr 30;10(4):e1001436. doi: 10.1371/journal.pmed.1001436 (PMC3640084; doi:10.1371/journal.pmed.1001436)
Supplement: Table S1 — Electronic search strategies. (PDF) [file pmed.1001436.s010.pdf]

Table S1 Electronic search strategies

Database	Search terms
Embase	1. Kidney Disease/<br>2. Kidney Failure/<br>3. Chronic Kidney Failure/<br>4. exp hemodialysis<br>5. (hemodialysis or haemodialysis).tw.<br>6. dialysis.tw.<br>7. (CAPD or CCPD or APD).tw.<br>8. predialysis.tw.<br>9. (chronic renal or chronic kidney).tw.<br>10. or/1-9<br>11. exp Bone Disease/<br>12. bone disease$.tw.<br>13. (bone$ and (atroph$ or formation or deform$ or destruct$ or necrosis or resorption or metabol$ or turnover or demineral$ or decalcif$ or density)).tw.<br>14. (osteo$ or hyperparathyroid$).tw.<br>15. Renal Osteodystrophy/<br>16. or/11-15<br>17. 10 and 16<br>18. Calcimimetic Agent/<br>19. Cinacalcet/<br>20. naphthalene derivative/ or naphthalene/<br>21. ("R-568" or "AMG 074" or "AMG 073" or "KRN 1493").tw.<br>22. calcimimetic$.tw.<br>23. cinacalcet.tw.<br>24. or/18-23<br>25. and/17,24
CENTRAL	1. KIDNEY DISEASES single term<br>2. KIDNEY FAILURE single term<br>3. KIDNEY FAILURE CHRONIC single term<br>4. RENAL DIALYSIS explode all trees<br>5. (hemodialysis or haemodialysis)<br>6. dialysis<br>7. (capd or ccpd or apd)<br>8. predialysis<br>9. ((chronic next renal) or (chronic next kidney))<br>10. (kidney next disease*)<br>11. (kidney next failure)<br>12. (#1 or #2 or #3 or #4 or #5 or #6 or #7 or #8 or #9 or #10 or #11)<br>13. BONE DISEASES explode all trees<br>14. RENAL OSTEODYSTROPHY single term<br>15. (bone next disease*)<br>16. (bone* and (atroph* or formation or deform* or destruct* or necrosis or resorption or metabol* or turnover or demineral* or decalcif* or density))<br>17. (osteo* or hyperparathyroid*)<br>18. (#13 or #14 or #15 or #16 or #17)<br>19. (#12 and #18)<br>20. calcimimetic*<br>21. cinacalcet<br>22. NAPTHTHALENES single term<br>23. (#20 or #21 or #22)<br>24. (#19 and #23)
