# Supplementary material for: Cinacalcet in Patients with Chronic Kidney Disease: A Cumulative Meta-Analysis of Randomized Controlled Trials
Source: PLoS Med. 2013 Apr 30;10(4):e1001436. doi: 10.1371/journal.pmed.1001436 (PMC3640084; doi:10.1371/journal.pmed.1001436)
Supplement: Table S2 — Included studies comparing cinacalcet plus conventional therapy versus placebo or no treatment plus conventional therapy in adults with chronic kidney disease. (PDF) [file pmed.1001436.s011.pdf]

Table S2 Included studies comparing cinacalcet plus conventional therapy versus placebo or no treatment plus conventional therapy in adults with chronic kidney disease

					Intervention (target PTH)		Non-randomised cointervention (type, %)					
**Study, year (reference)**	**Participants (treatment/ control)**	**Inclusion criteria**	**Exclusion criteria**	**Primary outcome**	**Cinacalcet (target serum PTH value)**	**Control (plus conventional care)**	**Cinacalcet**	**Control**	**Duration**	**Mean age, years**	**Stage of chronic kidney disease**	**Baseline parathyroid hormone level, pg/ml**
Goodman et al, 2000[19]	21 (16/5)	Hemodialysis ≥ 3 mo<br>Age ≥ 18 years<br>Serum PTH 300-1200 pg/ml<br>Serum calcium >9.0 mg/dl<br>Serum phosphorus >3.0 mg/dl	Women of childbearing age, hepatic transaminases or bilirubin twice ULN, medication including selective serotonin reuptake inhibitors, tricyclic antidepressants, beta-adrenergic blocking agents metabolized by cytochrome P450 pathway, seizures, malignancy, hyperthyroidism, granulomatous diseases causing hypercalcemia, myocardial infarction within previous 6 mo, corrected QT interval >450 milliseconds	Safety, efficacy and pharmacokinetics of repeated oral doses	R-568, 100 mg/d	Placebo	Vitamin D, 38%; phosphate binder, 31%	Vitamin D, 50%, phosphate binder, 50%	15 d maintenance, 24 d follow-up	Cinacalcet 48.6 (12.4)<br>Control 54.7 (16.8)	Hemodialysis	Approximately 600 pg/ml (figure format)
Goodman et al, 2002[20]	30 (23/7)	Hemodialysis ≥ 3 mo<br>iPTH, 250-1500 pg/ml<br>Serum calcium ≥9.0 mg/dl<br>Serum phosphorus ≥2.5 mg/dl	Women of childbearing age, hepatic transaminases or bilirubin twice ULN, seizures, malignancy within 5 y, hyperparathyroidism, myocardial infarction within 6 mo, cardiac ventricular rhythm disturbance, gastrointestinal disorder affecting absorption, granulomatous disease causing hypercalcaemia	Plasma PTH levels and calcium concentrations	AMG 073,* 10-50 mg/d	Placebo	Not available	Not available	8 d maintenance, 15 d follow-up	Not available	Hemodialysis	Cinacalcet, range 473 (284) to 919 (479)<br>Control 599 (362)
Lindberg et al, 2003[31]	78 (39/39)	Hemodialysis ≥ 3 mo<br>Serum PTH ≥300 pg/ml<br>Serum calcium ≥8.8 mg/dl and <11.0 mg/dl<br>Serum phosphorus ≥2.5 mg/dl<br>Serum calcium × phosphorus <70 mg/dl	No evidence of active infection, malignant process, or disease known to cause hypercalcemia. Hepatic transaminases or bilirubin twice ULN, hemoglobin >9.0 g/dl or hematocrit >27%	Reduction in PTH level ≥30%	AMG 073,* 10-50 mg/d (PTH ≥30% reduction from baseline)	Placebo	Vitamin D, 62%, phosphate binder, 87%	Vitamin D, 60%; phosphate binder, 87%	12 w titration, 6 w maintenance	Cinacalcet 52.7 (16.4)<br>Control 48.8 (15.6)	Hemodialysis	Cinacalcet 632 (280)<br>Control 637 (456)
Quarles et al, 2003[32]	71 (36/35)	Hemodialysis ≥ 3 mo<br>Serum PTH ≥300 pg/ml<br>Serum calcium ≥8.8 mg/dl and <11.0 mg/dl<br>Serum calcium × phosphorus <70 mg/dl	No evidence of active infection, malignant process, or disease known to cause hypercalcemia. Hepatic transaminases or bilirubin twice ULN, hemoglobin >9.0 g/dl or hematocrit >27%	Reduction in PTH level ≥30% from baseline	AMG 073,* 25-100 mg/d (PTH decrease ≥30% from baseline and absolute PTH ≤ 250 pg/ml	Placebo	Vitamin D, 61%; phosphate binder, 100%	Vitamin D, 69%; phosphate binder, 100%	12 w titration, 6 w maintenance	Cinacalcet 49.6 (8.5)<br>Control 47.9 (14.2)	Hemodialysis	Cinacalcet 451 (444)<br>Control 552 (484)
Block et al, 2004[33]	741 (371/370)	Hemodialysis ≥ 3 mo<br>iPTH ≥ 300 pg/ml<br>Serum calcium ≥ 8.4 mg/dl	Cancer, active infection, diseases known to cause hypercalcemia, receiving drugs such as flecainide, thioridazine, and tricyclic antidepressants	Mean PTH level of <250 pg/ml or less during efficacy treatment phase	Cinacalcet, 30-180 mg/d (iPTH ≤ 250 pg/ml)	Placebo	Vitamin D, 66%; phosphate binder, 92%	Vitamin D, 66%; phosphate binder, 92%	12 w titration, 14 w maintenance	Cinacalcet 54 (14)<br>Control 55 (15)	Hemodialysis	Cinacalcet 643 (347)<br>Control 642 (365)

					Intervention (target PTH)		Non-randomised cointervention (type, %)					
Study, year (reference)	Participants (treatment/ control)	Inclusion criteria	Exclusion criteria	Primary outcome	Cinacalcet (target serum PTH value)	Control (plus conventional care)	Cinacalcet	Control	Duration	Mean age, years	Stage of chronic kidney disease	Baseline parathyroid hormone level, pg/ml
Harris et al, 2004[34]	23 (17/6)	Hemodialysis<br>Serum calcium ≥ 8.4 mg/dl<br>Serum phosphorus ≥ 3.0 mg/dl	Not available	Pharmacokinetics, pharmacodynamics, safety and tolerability	Cinacalcet, 25 to 300 mg/d	Placebo	Vitamin D, 71%; phosphate binder, 100%	Vitamin D, 60%; phosphate binder, 80%	12 w titration	Cinacalcet 48.5 (10.4)<br>Control 58 (13.1)	Hemodialysis	Cinacalcet 330.9 (281.2)<br>Control 260.4 (194.5)
Charytan et al, 2005[35]	54 (27/27)	Chronic kidney disease (estimated glomerular filtration rate of 15 to 50 ml/min per 1.73 m^2<br>iPTH > 130 pg/ml<br>Serum calcium ≥ 9.0 mg/dl	Unstable medical condition, were pregnant or lactating, parathyroidectomy, myocardial infarction, kidney transplantation, changed vitamin D therapy, likely to begin dialysis or receive kidney transplant within 18 weeks	Reduction in PTH level ≥30% from baseline	Cinacalcet, 30 to 180 mg/d	Placebo	Vitamin D, 22%; phosphate binder, 37%	Vitamin D, 33%; phosphate binder, 48 %	12 w titration, 6 w maintenance	Cinacalcet 60.6 (15.6)<br>Control 61.9 (15.1)	Chronic kidney disease not treated with dialysis or transplant	Cinacalcet 243 (140)<br>Control 236 (187)
Lindberg et al, 2005[36]	395 (294/101)	Hemodialysis, continuous ambulatory peritoneal dialysis or automated peritoneal dialysis ≥ 1 mo<br>iPTH ≥ 300 pg/ml<br>Serum calcium ≥ 8.4 mg/dl	Unstable medical condition, parathyroidectomy, myocardial infarction within previous 3 mo	Mean iPTH level ≤250 pg/ml	Cinacalcet, 30 to 180 mg/d (iPTH ≤250 pg/ml)	Placebo	Not available	Not available	16 w titration, 10 w maintenance	Cinacalcet 51.8 (14)<br>Control 53.5 (13.9)	Hemodialysis or peritoneal dialysis	Cinacalcet 847.9 (688)<br>Control 832.1 (484)
ACHIEVE, 2008[37]	173 (87/86)	Hemodialysis > 3 mo receiving paricalcitol or doxercalciferol<br>PTH > 300 pg/ml<br>Serum calcium ≥ 8.4 mg/dl	Pregnant or nursing, parathyroidectomy within previous 3 mo, clinical trial within 30 d, or had cinacalcet	Simultaneous achievement of mean PTH between 150 and 300 pg/ml and a mean Ca ×P value <55 mg^2/dl^2	Cinacalcet, 30 to 180 mg/d plus paricalcitol 2 g or doxercalciferol 1 g IV thrice weekly (iPTH ≤ 250 pg/ml)	Paricalcitol 2 g or doxercalciferol 1 g IV thrice weekly (iPTH ≤ 250 pg/ml)	Vitamin D, 100%; phosphate binder, not available	Vitamin D, 100%; phosphate binder, not available	16 w titration, 11 w maintenance	Cinacalcet 57.7 (14.9)<br>Control 59 (12.4)	Hemodialysis	Cinacalcet† 597 (471, 775, Q1, Q3)<br>Control† 621 (463, 833, Q1, Q3)
Akiba et al, 2008[38]	121 (91/30)	Hemodialysis ≥ 12 weeks<br>Serum i PTH ≥ 300 pg/ml<br>Serum calcium 9.0 to 11.5 pg/ml	Severe hepatic disease, cirrhosis, severe heart failure, uncontrolled hypertension, uncontrolled diabetes mellitus, malignancy, serious infection, pregnancy or lactation, parathyroidectomy within 24 w or parathyroid intervention therapies	Percent change from baseline in iPTH levels	Cinacalcet, 12.5 to 50 mg/d	Placebo	Vitamin D, 63-74%; phosphate binder, 87.5-100%	Vitamin D, 70%; phosphate binder, 100%	3 w maintenance, 2 w follow-up	Cinacalcet 12.5 mg/d 56.7 (9.2)<br>25 mg/d 55.8 (7.7)<br>50 mg/d 53.2 (7.0)<br>Control 51.8 (7.5)	Hemodialysis	Cinacalcet† 12.5 mg 705(515-800, Q1, Q3)<br>25 mg 630 (459-769, Q1, Q3)<br>50 mg 642 (514-842, Q1, Q3)<br>Control† 601 (515-846, Q1, Q3)
Fugakawa et al, 2008[39]	143 (72/71)	Hemodialysis ≥ 16 w<br>iPTH ≥ 300 pg/ml<br>Serum calcium ≥ 9.0 mg/dl	Parathyroidectomy within 24 w, percutaneous ethanol injection into parathyroid during screening, severe hepatic disease, severe hypertension, uncontrolled diabetes, cancer, severe infection, severe heart failure	Serum iPTH ≤250 pg/ml	Cinacalcet, 30 to 180 mg/d (iPTH ≤ 250 pg/ml)	Placebo	Vitamin D, 88%; phosphate binder, 93%	Vitamin D, 89%; phosphate binder, 96%	14 w maintenance	Cinacalcet 54.7 (11)<br>Control 55.7 (11.7)	Hemodialysis	Cinacalcet† 607(439-849, Q1, Q3)<br>Control† 552 (420-864, Q1, Q3)

					Intervention (target PTH)		Non-randomised cointervention (type, %)					
**Study, year (reference)**	**Participants (treatment/ control)**	**Inclusion criteria**	**Exclusion criteria**	**Primary outcome**	**Cinacalcet (target serum PTH value)**	**Control (plus conventional care)**	**Cinacalcet**	**Control**	**Duration**	**Mean age, years**	**Stage of chronic kidney disease**	**Baseline parathyroid hormone level, pg/ml**
Malluche et al, 2008[40]	48 (32/16)	Hemodialysis ≥ 1 mo<br>Biochemical secondary hyperparathyroidism<br>Serum calcium ≥ 8.4 mg/dl	Bisphosphonate or fluoride therapy within 90 d	Bone histomorphometry	Cinacalcet 30 to 180 mg/d (iPTH ≤200 pg/ml)	Placebo	Vitamin D, 47%; phosphate binder, 100%	Vitamin D, 54%; phosphate binder, 77%	24 w titration<br>28 w maintenance	Cinacalcet<br>50.3 (13.3)<br>Control<br>51.5 (14.1)	Hemodialysis	Cinacalcet<br>713 (399)<br>Control<br>703 (378)
OPTIMA 2008[41]	552 (368/184)	Hemodialysis ≥ 1 mo<br>iPTH ≥ 300 pg/ml and <800 pg/ml<br>Serum calcium ≥ 8.4 mg/dl	Unstable medical condition, breastfeeding, myocardial infarction within 3 mo, parathyroidectomy within 6 mo, gastrointestinal disorder, vitamin D therapy for <21 d, enrolled in other study or cinacalcet trial	Mean iPTH ≤300 pg/ml during efficacy phase	Cinacalcet 30 to 180 mg/d (iPTH ≤ 300 pg/ml)	No treatment	Vitamin D, 68%; phosphate binder, 92%	Vitamin D, 68%; phosphate binder, 90%	16 w titration<br>7 w maintenance	Cinacalcet<br>58.5 (14.5)<br>Control<br>58.3 (14.5)	Hemodialysis	Cinacalcet<br>505 (147)<br>Control<br>507 (143)
Chonchol et al, 2009[42]	404 (302/102)	Chronic kidney disease stage 3 or 4 (estimated glomerular filtration rate 15 to 59 ml/min per 1.73 m^2<br>iPTH ≥100 pg/ml (stage 3) or ≥ 160 pg/ml<br>Serum calcium ≥9.0 mg/dl	Kidney transplantation, pregnancy, lactation, primary hyperparathyroidism, unstable medical condition, participation in previous cinacalcet trial, likelihood of dialysis or scheduled kidney transplantation within 28 w of enrolment, myocardial infarction within 3 mo before enrollment, participation in another investigational trial, prior treatment with cinacalcet, change in active vitamin D sterol treatment in previous 30 days	Reduction in PTH level ≥30% from baseline	Cinacalcet 30 to 180 mg/d (iPTH ≤70 pg/ml for stage 3 CKD and ≤110 pg/ml for stage 4 CKD	Placebo	Vitamin D, 21%; phosphate binder, 19%	Vitamin D, 21%; phosphate binder, 18%	16 w titration<br>16 w maintenance	Cinacalcet<br>64.7 (13.3)<br>Control<br>66.2 (12.2)	Chronic kidney disease not treated with dialysis or transplant	Cinacalcet<br>262 (168)<br>Control<br>269 (179)
ADVANCE, 2011[43]	360 (180/180)	Hemodialysis ≥ 3 mo<br>iPTH >300 pg/ml<br>Serum calcium ≥ 8.4 mg/dl<br>Serum calcium × phosphorus >50 mg^2/dl^2	Previous cinacalcet treatment, calcium-free phosphate binding agents, bisphosphonate therapy, lipid lowering within 30 d, atrial fibrillation, coronary artery bypass grafting or stent, valve replacement, heart transplant, pacemaker, aortic aneurysm, parathyroidectomy within 3 mo or in next 6 mo, scheduled kidney transplant, body weight >136 kg, inability to absorb oral medications, sensitivity to cinacalcet, unstable medical condition	Percent change in Agatston coronary artery calcification score from baseline to end of treatment	Cinacalcet 30 to 180 mg/d plus low dose vitamin D therapy (IV calcitriol 0.5 µg, alfacalcidol 1 µg, doxercalciferol 1 µg, or paricalcitol 2 µg paricalcitol with each dialysis or PO as calcitriol 0.25 µg alternate days or alfacalcidol 0.25 µg d) (iPTH <300 pg/ml)	Same dose of vitamin D prescribed before randomization	Vitamin D, 75%; phosphate binder, (calcium based 83%; sevelamer 26%; lanthanum 4%; other 11%)	Vitamin D, 79%; phosphate binder, (calcium-based 84%; sevelamer 26%; lanthanum 7%; other 8%)	20 w titration<br>32 w maintenance	Cinacalcet<br>61.2 (12.6)<br>Control<br>61.8 (12.8)	Hemodialysis	Cinacalcet†<br>432 (243-1056, 10th-90th percentile)<br>Control†<br>424 (257-1176, 10th-90th percentile)

					Intervention (target PTH)		Non-randomised cointervention (type, %)					
**Study, year (reference)**	**Participants (treatment/ control)**	**Inclusion criteria**	**Exclusion criteria**	**Primary outcome**	**Cinacalcet (target serum PTH value)**	**Control (plus conventional care)**	**Cinacalcet**	**Control**	**Duration**	**Mean age, years**	**Stage of chronic kidney disease**	**Baseline parathyroid hormone level, pg/ml**
El-Shafey et al, 2011[44]	82 (55/27)	Hemodialysis ≥ 3 mo<br>iPTH ≥279 pg/ml<br>Serum calcium ≥8.4 mg/dl	Unstable medical condition, breastfeeding, myocardial infarction within 3 mo, parathyroidectomy within 6 mo, impaired absorption of oral medications, vitamin D therapy <21 d, change in prescribed vitamin D agent or dose within 21 d, other studies or previously enrolled in cinacalcet trial	iPTH ≤31.8 pmol/l (279 pg/ml)	Cinacalcet 30 to 180 mg/d (iPTH<279 pg/ml)	No treatment	Vitamin D, 50%; phosphate binder, 86%	Vitamin D, 52%; phosphate binder, 89%	12 w titration<br>24 w maintenance	Cinacalcet<br>51.5 (12.7)<br>Control<br>51.8 (15.0)	Hemodialysis	Cinacalcet 619 (149)<br>Control<br>657 (170)
IMPACT SHPT study., 2012[45]	268 (134/134)	Hemodialysis ≥ 3 mo<br>iPTH 130-700 pg/ml<br>Serum calcium ≤ 10.0 mg/dl<br>Serum calcium × phosphorus ≤ 75 mg^2/dl^2 in United States centers<br>Serum calcium × phosphorus ≤ 75 mg^2/dl^2 in non-United States centers	Allergic reaction or significant insensitivity to any study drug, an expected daily requirement >2.0 g oral elemental calcium, previous parathyroidectomy, chronic gastrointestinal disorder, clinically significant liver disease, use of known inhibitors or inducers of cytochrome P450 3A or of drugs metabolized by cytochrome P450 2D6 within 2 w	iPTH 150-300 pg/ml during evaluation period	Cinacalcet (dose unclear) plus low dose vitamin D (doxercalcife rol 1.0 μg IV thrice weekly (US) or alfacalcidol 0.25 μg/d (non-US) (iPTH 150-300 pg/ml)	Paricalcitol 0.07 μg/kg IV (US + Russia) or iPTH/60 PO (other sites) (frequency of drug unclear)	Unclear	Unclear	28 w	Cinacalcet<br>59.9 (12.0)<br>US and Russia<br>65.1 (12.5)<br>other sites<br>Control<br>61.2 (12.7)<br>US and Russia<br>65.7 (13.5)<br>other sites	Hemodialysis	Cinacalcet<br>521 (149) US and Russia<br>510 (139) other sites<br>Control<br>526 (153) US and Russia<br>495 (170) other sites
EVOLVE 2012[23]	3883 (1948/1935)	Hemodialysis > 3 mo<br>iPTH ≥ 300 pg/ml<br>Serum calcium ≥8.4 mg/dl<br>Serum calcium × phosphorus >45 mg^2/dl^2	Unstable medical condition, parathyroidectomy within 12 w, severe concomitant disease, cinacalcet within 3 mo, hospitalization within 12 w for myocardial infarction, unstable angina, heart failure, peripheral vascular disease, stroke, history of seizure within 12 w, scheduled kidney transplant, anticipated parathyroidectomy within 6 mo, other investigational procedures, intolerance to calcimimetic, unable to give consent, pregnancy, breast feeding or childbearing potential with inadequate contraception	Composite of time to all-cause mortality or first nonfatal cardiovascular event (myocardial infarction, hospitalization for unstable angina, heart failure, or peripheral vascular disease, including lower extremity revascularization and nontraumatic amputation)	Cinacalcet 30 to 180 mg/d (iPTH 150-300 pg/ml)	Placebo	Vitamin D, 59.3%; phosphate binder, 87.8%	Vitamin D, 59.6%; phosphate binder, 89.0%	20 w titration	Cinacalcet†<br>55.0<br>(35-74 10th-90th percentile)<br>Control†<br>54.0<br>(35-73 10th – 90th percentile)	Hemodialysis	Cinacalcet† 695 (362-1707 10th-90th percentile)<br>Control†<br>690 (363-1683$10^{th}$-90th percentile)

R-568, AMG073 and cinacalcet are the same compound.

†Median
