# Supplementary material for: Cinacalcet in Patients with Chronic Kidney Disease: A Cumulative Meta-Analysis of Randomized Controlled Trials
Source: PLoS Med. 2013 Apr 30;10(4):e1001436. doi: 10.1371/journal.pmed.1001436 (PMC3640084; doi:10.1371/journal.pmed.1001436)
Supplement: Table S3 — Definitions of parathyroid hormone and calcium targets triggering reduction in cinacalcet dose, and definition of hypocalcemia and hypercalcemia end points in included trials. (PDF) [file pmed.1001436.s012.pdf]

Table S3 Definitions of parathyroid hormone and calcium targets triggering reduction in cinacalcet dose and definition of hypocalcemia and hypercalcemia end points in included trials

Study, year (reference)	Participants (treatment/ control)	Parathyroid hormone level triggering reduction in cinacalcet dose	Calcium level triggering reduction in cinacalcet dose	Hypocalcemia (study endpoint)	Hypercalcemia (study endpoint)
Goodman et al, 2000[19]	21 (16/5)	…	Symptoms of hypocalcemia or ionized calcium <4 mg/dl	Ionized calcium <4 mg/dl	…
Goodman et al, 2002[20]	30 (23/7)	…	8.0 mg/dl	<8.0 mg/dl	…
Lindberg et al, 2003[31]	78 (39/39)	<100 pg/ml	Symptoms of hypocalcemia or <7.8 mg/dl	<7.5 mg/dl	…
Quarles et al, 2003[32]	71 (36/35)	<100 pg/ml	<7.8 mg/dl	…	…
Block et al, 2004[33]	741 (371/370)	<100 pg/ml	Symptoms of hypocalcemia or <7.8 mg/dl	Withdrawal due to hypocalcemia	…
Harris et al, 2004[34]	23 (17/6)	…	…	…	…
Charytan et al, 2005[35]	54 (27/27)	…	Dose-related adverse event or <7.8 mg/dl	<8.4 mg/dl	…
Lindberg et al, 2005[36]	395 (294/101)	…	Symptoms of hypocalcemia or <7.8 mg/dl	…	…
ACHIEVE, 2008[37]	173 (87/86)	<150 pg/ml	Symptoms of hypocalcemia or <7.5 mg/dl	<8.4 mg/dl	>10.2 mg/dl
Akiba et al, 2008[38]	121 (91/30)	…	…	Hypocalcemia	…
Fugakawa et al, 2008[39]	145 (72/73)	Investigators' discretion or excessive decrease in PTH level	Investigators' discretion or <7.5 mg/dl	Hypocalcemia	…
Malluche et al, 2008[40]	32 (19/13)	<100 pg/ml	Symptoms of hypocalcemia or <7.8 mg/dl	…	…
OPTIMA ,2008[41]	552 (368/184)	<150 pg/ml	<8.0 mg/dl	<7.5 mg/dl	…
Chonchol et al, 2009[42]	404 (302/102)	PTH <35 pg/ml for stage 3 and <70 pg/ml for stage 4	Symptoms of hypocalcemia or <7.5 mg/dl	<7.5 mg/dl	…
ADVANCE, 2011[43]	360 (180/180)	…	…	Hypocalcemia	Hypercalcemia
El-Shafey et al, 2011[44]	82 (55/27)	<92 pg/ml	Dose-related adverse event or <7.5 mg/dl	Hypocalcemia	…
IMPACT SHPT, 2012[45]	264 (134/134)	<150 pg/ml	<7.5 mg/dl	<8,.4 mg/dl	>10.5 mg/dl
EVOLVE, 2012 [23]	3883 (1948/1935)	<150 pg/ml	<7.5 mg/dl and/or symptoms of hypocalcemia	<8.0 mg/dl or <7.5 mg/dl (unclear which threshold reported in study)	>10.5 mg/dl
