# Supplementary material for: Cinacalcet in Patients with Chronic Kidney Disease: A Cumulative Meta-Analysis of Randomized Controlled Trials
Source: PLoS Med. 2013 Apr 30;10(4):e1001436. doi: 10.1371/journal.pmed.1001436 (PMC3640084; doi:10.1371/journal.pmed.1001436)
Supplement: Table S4 — Effects of cinacalcet plus conventional therapy versus placebo or no treatment plus conventional therapy on end-of-treatment serum parathyroid hormone, phosphorus, and calcium concentrations in adults with chronic kidney disease. (PDF) [file pmed.1001436.s013.pdf]

Table S4 Effects of cinacalcet plus conventional therapy versus placebo or no treatment plus conventional therapy on end of treatment serum parathyroid hormone, phosphorus and calcium concentrations in adults with chronic kidney disease

**Outcome**	**Studies/ Participants, *n/N***	**Random-Effects Mean Difference (95% CI)**	**I^2, %**
Serum parathyroid hormone	7/1935	-281 pg/ml (-326 to -236)	33
Serum phosphorus	8/2300	-0.23 mg/dl (-0.58 to 0.12)	88
Serum calcium	6/1004	-0.89 mg/dl (-1.01 to -0.77)	18
