# Supplementary material for: Cinacalcet in Patients with Chronic Kidney Disease: A Cumulative Meta-Analysis of Randomized Controlled Trials
Source: PLoS Med. 2013 Apr 30;10(4):e1001436. doi: 10.1371/journal.pmed.1001436 (PMC3640084; doi:10.1371/journal.pmed.1001436)
Supplement: Table S5 — Univariate metaregression exploring the role of patient and trial characteristics in the effect of cinacalcet therapy on clinical outcomes. (PDF) [file pmed.1001436.s014.pdf]

**Table S5 Univariate meta-regression exploring the role of patient and trial characteristics on the effect of cinacalcet therapy on clinical outcomes**

| Covariate | No. of studies reporting 1 or more events | Scale | Proportional change in risk ratio (95% CI) | P value |
|---|---|---|---|---|
| ***All-cause mortality*** | | | | |
| Mean trial age | 11 | Per 1-year increase | -0.03 (-0.11 to 0.06) | 0.49 |
| Proportion of male participants | 10 | Per 1% increase | -0.01 (-0.15 to 0.12) | 0.84 |
| Baseline serum PTH concentration | 10 | Per 100 pg/ml increase | 0.07 (-0.13 to 0.28) | 0.48 |
| Baseline serum calcium concentration | 10 | Per 1 mg/dl increase | -0.03 (-1.85 to 1.78) | 0.96 |
| Trial duration | 11 | Per 1 month increase | 0.02 (-0.01 to 0.06) | 0.25 |
| Year of publication | 11 | Per 1-year increase | 0.05 (-0.05 to 0.14) | 0.36 |
| ***Parathyroidectomy*** | | | | |
| Mean trial age | 5 | Per 1-year increase | 0.12 (-0.22 to 0.46) | 0.49 |
| Proportion of male participants | 5 | Per 1% increase | -0.48 (-1.02 to 0.06) | 0.09 |
| Baseline serum PTH concentration | 5 | Per 100 pg/ml increase | 0.15 (-0.74 to 1.06) | 0.72 |
| Baseline serum calcium concentration | 5 | Per 1 mg/dl increase | 3.05 (-1.15 to 7.30) | 0.16 |
| Trial duration | 5 | Per 1 month increase | 0.11 (-0.01 to 0.25) | 0.08 |
| Year of publication | 5 | Per 1-year increase | 0.28 (-0.09 to 0.66) | 0.13 |
| ***Hypocalcemia*** | | | | |
| Mean trial age | 13 | Per 1-year increase | 0.15 (0.02 to 0.29) | 0.02 |
| Proportion of male participants | 13 | Per 1% increase | 0.00 (-0.10 to 0.10) | 0.94 |
| Baseline serum PTH concentration | 12 | Per 100 pg/ml increase | -0.26 (-0.59 to 0.06) | 0.11 |
| Baseline serum calcium concentration | 12 | Per 1 mg/dl increase | -0.78 (-4.00 to 2.45) | 0.64 |
| Trial duration | 14 | Per 1 month increase | 0.00 (-0.05 to 0.05) | 0.92 |
| Year of publication | 14 | Per 1-year increase | 0.05 (-0.05 to 0.15) | 0.38 |
| Serum calcium level used to define hypocalcemia | 8 | Per 1 mg/dl increase | 1.96 (0.31 to 3.62) | 0.02 |
| ***Nausea*** | | | | |
| Mean trial age | 13 | Per 1-year increase | 0.05 (0.01 to 0.10) | 0.02 |
| Proportion of male participants | 13 | Per 1% increase | -0.01 (-0.08 to 0.05) | 0.62 |
| Baseline serum PTH concentration | 13 | Per 100 pg/ml increase | -0.10 (-0.21 to 0.00) | 0.05 |
| Baseline serum calcium concentration | 12 | Per 1 mg/dl increase | -0.62 (-1.75 to 0.52) | 0.28 |
| Trial duration | 14 | Per 1 month increase | 0.00 (-0.01 to 0.02) | 0.74 |
| Year of publication | 14 | Per 1-year increase | 0.02 (-0.01 to 0.05) | 0.19 |
