# Supplementary material for: Cinacalcet in Patients with Chronic Kidney Disease: A Cumulative Meta-Analysis of Randomized Controlled Trials
Source: PLoS Med. 2013 Apr 30;10(4):e1001436. doi: 10.1371/journal.pmed.1001436 (PMC3640084; doi:10.1371/journal.pmed.1001436)
Supplement: Text S2 — Protocol. (PDF) [file pmed.1001436.s016.pdf]

Calcimimetics for secondary hyperparathyroidism in chronic kidney disease patients

Review information

Review number: 085

Authors

Giovanni FM Strippoli[1], Allison Tong[2], Suetonia C Palmer[3], Grahame J Elder[4], Jonathan C Craig[5]

[1]Department of Clinical Pharmacology and Epidemiology, Mario Negri Sud Consortium, Santa Maria Imbaro, Italy

[2]Centre for Kidney Research, The Children's Hospital at Westmead, Westmead, Australia

[3]Department of Medicine, University of Otago Christchurch, Christchurch, New Zealand

[4]a) Department of Renal Medicine, Westmead Hospital, b) Osteoporosis and Bone Biology Program, Garvan Institute of Medical Research, Darlinghurst, Australia

[5]Cochrane Renal Group, Centre for Kidney Research, The Children's Hospital at Westmead, Westmead, Australia

Citation example: Strippoli GFM, Tong A, Palmer SC, Elder GJ, Craig JC. Calcimimetics for secondary hyperparathyroidism in chronic kidney disease patients. Cochrane Database of Systematic Reviews 2006, Issue 4. Art. No.: CD006254. DOI: 10.1002/14651858.CD006254.

Contact person

Giovanni FM Strippoli

Cochrane Renal Group, Centre for Kidney Research
The Children's Hospital at Westmead
Locked Bag 4001
Westmead
NSW
2145
Australia

E-mail: strippoli@negrisud.it

Dates

**Assessed as Up-to-date:**	12 July 2006
**Date of Search:**	12 July 2006
**Next Stage Expected:**	14 June 2007
**Protocol First Published:**	Issue 4, 2006
**Review First Published:**	Issue 4, 2006
**Last Citation Issue:**	Issue 4, 2006

What's new

Date / Event	Description
01 November 2011 Amended	Minor changes to protocol
14 January 2010 Amended	Contact details updated.

History

Date / Event	Description
13 August 2009 Amended	Contact details updated.
13 May 2009 Amended	Contact details updated.
16 September 2008 Amended	Converted to new review format.

Abstract

Background

Objectives

Search methods

Selection criteria

Data collection and analysis

Results

Authors' conclusions

Plain language summary

Background

Abnormalities of calcium and phosphorus metabolism are present in all individuals with chronic kidney disease (CKD). These biochemical changes cause many bone and metabolic disorders, including renal osteodystrophy that is characterized by abnormalities of bone turnover (ranging from high turnover osteitis fibrosa to adynamic bone disease), mineralization defects (osteomalacia) and architectural change. Renal osteodystrophy is associated not only with an increased incidence of fracture, bone and muscular pain and abnormalities of bone and joint morphology, but also with vascular and soft tissue calcification. In addition to causing reduced quality of life, these complications and the associated abnormalities of elevated phosphorus, calcium, the calcium by phosphorus product and levels of parathyroid hormone (PTH) have been associated with increased mortality (Block 2004b; Ganesh 2001; Malluche 2004b; Marco 2003; Martin 2004; Stehman-Breen 2004).

Standard management of patients with CKD, particularly those on dialysis, includes treatment to control levels of calcium, phosphorus and PTH, so as to prevent bone and soft-tissue complications. Based on a number of association studies, (Block 2004b; Ganesh 2001; Kestenbaum 2005; Marco 2003; Stevens 2004) including studies of bone histomorphometry, (Hutchison 1993; Qi 1995; Wang 1995; Ziolkowska 2000) optimal ranges for serum phosphorus, calcium, the calcium by phosphorus product and PTH have been suggested (CARI 2005; NKF 2003). However, success in achieving these targets has been limited (Young 2004).

Specific management of secondary hyperparathyroidism (SHPT) in CKD stages 3 and 4 may be accomplished by restriction of dietary phosphorus, calcium supplementation, and/or the use of calcitriol. Once patients have commenced dialysis, standard therapy of SHPT generally includes calcitriol, vitamin D analogues or derivatives, calcium or other phosphate-binding agents and parathyroidectomy (Albaaj 2003; Courant 1993). Recently the use of a novel class of drugs, the calcimimetics, has been proposed as a strategy to reduce PTH secretion and possibly to reduce parathyroid cell proliferation, while decreasing levels of serum calcium, phosphorus and the calcium by phosphorus product (Ott 1998). Use of these agents has been advocated whenever there is inability to control SHPT with other agents. Results of randomised controlled trials (RCTs) testing the efficacy and safety of calcimimetics in patients undergoing dialysis are becoming available. With the aim of preventing complications associated with SHPT, cinacalcet HCl has now been incorporated into many treatment algorithms.

However, several aspects of calcimimetic therapy require further evaluation. In the dialysis population, for which these drugs are approved, the most important question is the degree to which calcimimetics will impact on clinically relevant end-points such as parathyroidectomy rates, fracture, renal osteodystrophy, cardiovascular disease and death, as well as surrogate markers for these conditions, such as abnormalities of serum calcium and phosphorus. Other important questions include the optimal time for commencement of calcimimetic therapy, the influence of calcimimetics on standard treatment regimens and the effectiveness of calcimimetics at different stages of CKD and after transplantation.

This systematic review will review the evidence for benefits and harms of calcimimetic therapy in adults with chronic kidney disease.

Objectives

To evaluate the benefits and harms of calcimimetics for the prevention of secondary hyperparathyroid bone disease (including osteitis fibrosa cystica and adynamic bone disease) in patients with CKD.

Methods

Criteria for considering studies for this review

Types of studies

We will include RCTs of any calcimimetic agent, cinacalcet HCl (AMG-073, Sensipar®), NPS R-467 or NPS R-568 administered to patients with CKD for the treatment of SHPT.

Types of participants

Patients with CKD needing treatment for SHPT.

Types of interventions

Any calcimimetic agent (e.g. cinacalcet HCl (AMG-073, Sensipar®), NPS R-467 or NPS R-568).

Types of outcome measures

1. All-cause mortality
2. At least 30% decrease in mean PTH level
3. Fractures
4. Hypocalcaemia (as defined by the authors)
5. Nausea
6. Vomiting
7. Dyspnoea
8. Muscle weakness
9. Hypotension
10. Upper respiratory tract infection
11. Parathyroidectomy
12. Headache
13. Paraesthesia
14. Abdominal pain
15. Diarrhoea
16. Mixed uraemic osteodystrophy
17. Bone histomorphometry
18. End of treatment PTH levels (any measure)
19. End of treatment serum calcium concentrations (mg/dL or mmol/L)
20. End of treatment serum phosphorus concentrations (mg/dL or mmol/L)
21. End of treatment calcium x phosphorus product (mg^2/dL^2)

Search methods for identification of studies

The literature searching will be performed independently by two authors. Relevant studies will be obtained from the following sources without language restriction (see Table 1 - *Electronic search strategies*).

1. The Cochrane Renal Group's specialised register and the Cochrane Central Register of Controlled Trials (CENTRAL) in T*he Cochrane Library*
2. EMBASE using a search strategy adapted from that developed for the Cochrane Collaboration for the identification of RCTs (Lefebvre 1996) together with a specific search strategy developed with input from the Cochrane Renal Group's Trial Search Co-ordinator.
3. Reference lists of nephrology textbooks, review articles and relevant trials.
4. Letters seeking information about unpublished or incomplete trials to investigators known to be involved in previous trials.
5. Personal records and citation alerts

The Specialised Register contains studies identified from the Cochrane Central Register of Controlled Trials (CENTRAL), MEDLINE, handsearching of kidney-related journals and proceedings of major conferences, and searches of trials registries using search strategies based on the scope of the Cochrane Renal Group

(http://www.cochrane-renal.org/crgtopics.php).

Data collection and analysis

Selection of studies

The results of the electronic searches will be analysed in title and abstract form by two authors according to the inclusion criteria in consultation with a third author. We will consider all randomised controlled trials of any calcimimetic agent (cinacalcet HCl, NPS R-467, or NPS R568) and that report data for adults with CKD (any stage). Reference lists from the identified articles will also be searched and information about unpublished or ongoing trials will be sought from experts in the field and pharmaceutical companies. Trials will be considered without language restriction.

Data extraction and management

Each trial will be assessed independently by two authors. From all included trials, data will be extracted on study sample characteristics, the type of agent, dose, and route of administration, the trial methods and outcomes. Discrepancies in data extraction will be resolved by discussion among the authors, and when data were missing or incomplete, the investigators of the trial will be contacted for clarification.

Assessment of risk of bias in included studies

The methods and quality of included trials will be assessed using standard criteria (sequence generation, allocation concealment, blinding of participants and personnel, blinding of outcome assessment, analysis by intention to treat and completeness of outcome data, selective reporting, and other sources of bias).

Sequence generation

- Low risk: The investigators describe a random component in the sequence generation process
- High risk: The investigators describe a non-random component in the sequence generation process

Allocation concealment

- Low risk: Randomisation method described that would not allow investigator/participant to know or influence intervention group before eligible participant entered in the study
- Unclear risk: Randomisation stated but no information on method used is available
- High risk: Method of randomisation used such as alternate medical record numbers or unsealed envelopes; any information in the study that indicated that investigators or participants could influence intervention group

Blinding

- Blinding of investigators: Yes/no/not stated
- Blinding of participants: Yes/no/not stated
- Blinding of outcome assessor: Yes/no/not stated
- Blinding of data analysis: Yes/no/not stated

The above are considered not blinded if the treatment group can be identified in > 20% of participants because of the side effects of treatment.

Intention-to-treat

- Yes: Specifically reported by authors that intention-to-treat analysis was undertaken and this was confirmed on study assessment.
- Yes: Not stated but confirmed on study assessment

- No: Not reported and lack of intention-to-treat analysis confirmed on study assessment. (Patients who were randomised were not included in the analysis because they did not receive the study intervention, they withdrew from the study or were not included because of protocol violation)
- No: Stated but not confirmed upon study assessment
- Not stated

Incomplete outcome data

- Per cent of participants excluded or lost to follow-up.

Selective reporting

- Low risk: The study protocol is available and all the the study's pre-specified outcomes have been reported or the study protocol is not available but it is clear from published reports that all expected outcomes are reported
- High risk: Not all study pre-specified primary outcomes have been reported or the study report fails to include results for a key outcome that would have been expected to have been reported for such a study

Other sources of bias

- Low risk: The study appears to be free of other sources of bias
- High risk: There is at least one important risk of bias

Measures of treatment effect

The estimate of effect of an experimental versus a control intervention on categorical outcomes (e.g. fracture rate, all-cause mortality including sudden death) will be analysed using the risk ratio (RR) measure and its 95% confidence interval (CI) for each trial. For continuous variables, the mean difference (MD), and its 95% CI will be calculated using the end of treatment values of the variable in the experimental and control groups. Data will be summarised using standard and cumulative random-effects meta-analysis (based on year or publication).

Assessment of heterogeneity

Heterogeneity of treatment effects between studies will be tested formally using the Q (heterogeneity chi-square) and the I^2 statistic (Higgins 2003). We will consider a *P* value below 0.10 to indicate significant heterogeneity.

Assessment of reporting biases

To assess potential bias from small study effects, funnel plots for the log risk ratio in individual studies against the SE of the risk ratio will be generated and formally assessed for asymmetry by using the Egger regression test.(Egger 1998)

Data synthesis

Data will be pooled using a random effects model. For each analysis, the fixed effects model will also be evaluated to ensure robustness of the model chosen and susceptibility to outliers.

Subgroup analysis and investigation of heterogeneity

Additional pre-specified subgroup analyses and univariate random-effects meta-regression will be performed to explore potential sources of heterogeneity in treatment effects on all-cause mortality, parathyroidectomy, hypocalcaemia and nausea. We will evaluate the effects of age, proportion of male participants, baseline serum PTH concentration, baseline serum calcium concentration, trial duration, allocation concealment (adequate *versus* unclear) and year of publication. In addition, for the outcome of hypocalcaemia we will evaluate the serum calcium concentration used to define one or more hypocalcaemia events as a source of

heterogeneity in treatment effects for this outcome.

Sensitivity analysis

We will analyse data excluding trials in which randomised co-interventions (vitamin D compounds) are not equal between study groups. We will also restrict analyses to studies in which follow up duration was 6 months or longer.

Results

Description of studies

Risk of bias in included studies

Effects of interventions

Discussion

Authors' conclusions

Implications for practice

Implications for research

Acknowledgements

Contributions of authors

Declarations of interest

Differences between protocol and review

The protocol has been updated since first publication in 2006. The updated protocol now includes additional risk of bias items (sequence generation, selective outcome reporting and other sources of bias) according to The Cochrane Collaboration's standardised methods. The protocol now also includes pre-specified subgroup and univariate meta-regression analyses to explore for sources of heterogeneity between treatment estimates and additional sensitivity analyses. The electronic search strategies now exclude searches in Ovid MEDLINE, as these citations are included in searches of the Cochrane Renal Group's specialised register.

Published notes

Characteristics of studies

Characteristics of included studies

Footnotes

Characteristics of excluded studies

Footnotes

Characteristics of studies awaiting classification

Footnotes

Characteristics of ongoing studies

Footnotes

Summary of findings tables

Additional tables

1 Electronic search strategies

Database	Search terms
CENTRAL	1. KIDNEY DISEASES single term<br>2. KIDNEY FAILURE single term<br>3. KIDNEY FAILURE CHRONIC single term<br>4. RENAL DIALYSIS explode all trees<br>5. (hemodialysis or haemodialysis)<br>6. dialysis<br>7. (capd or ccpd or apd)<br>8. predialysis<br>9. ((chronic next renal) or (chronic next kidney))<br>10. (kidney next disease*)<br>11. (kidney next failure)<br>12. (#1 or #2 or #3 or #4 or #5 or #6 or #7 or #8 or #9 or #10 or #11)<br>13. BONE DISEASES explode all trees<br>14. RENAL OSTEODYSTROPHY single term<br>15. (bone next disease*)<br>16. (bone* and (atroph* or formation or deform* or destruct* or necrosis or resorption or metabol* or turnover or demineral* or decalcif* or density))<br>17. (osteo* or hyperparathyroid*)<br>18. (#13 or #14 or #15 or #16 or #17)

	19. (#12 and #18)<br>20. calcimimetic*<br>21. cinacalcet<br>22. NAPHTHALENES single term<br>23. (#20 or #21 or #22)<br>24. (#19 and #23)
EMBASE	1. Kidney Disease/<br>2. Kidney Failure/<br>3. Chronic Kidney Failure/<br>4. exp hemodialysis/<br>5. (hemodialysis or haemodialysis).tw.<br>6. dialysis.tw.<br>7. (CAPD or CCPD or APD).tw.<br>8. predialysis.tw.<br>9. (chronic renal or chronic kidney).tw.<br>10. or/1-9<br>11. exp Bone Disease/<br>12. bone disease$.tw.<br>13. (bone$ and (atroph$ or formation or deform$ or destruct$ or necrosis or resorption or metabol$ or turnover or demineral$ or decalcif$ or density)).tw.<br>14. (osteo$ or hyperparathyroid$).tw.<br>15. Renal Osteodystrophy/<br>16. or/11-15<br>17. 10 and 16<br>18. Calcimimetic Agent/<br>19. Cinacalcet/<br>20. naphthalene derivative/ or naphthalene/<br>21. ("R-568" or "AMG 074" or "AMG 073" or "KRN 1493").tw.<br>22. calcimimetic$.tw.<br>23. cinacalcet.tw.<br>24. or/18-23<br>25. and/17,24

Footnotes

References to studies

Included studies

Excluded studies

Studies awaiting classification

Ongoing studies

Other references

Additional references

Albaaj 2003

Albaaj F, Hutchison A. Hyperphosphataemia in renal failure: causes, consequences and current management. Drugs 2003;63(6):577-96. [MEDLINE: 12656655]

ANZDATA 2004

Australia and New Zealand Dialysis and Transplant Registry (ANZDATA). ANZDATA 27th Annual Report. http://www.anzdata.org.au/anzdata/AnzdataReport/download.htm (accessed August 8 2005).

Avram 1996

Avram MM, Sreedhara R, Avram DK, Muchnick RA, Fein P. Enrollment parathyroid hormone level is a new marker of survival in hemodialysis and peritoneal dialysis therapy for uremia. American Journal of Kidney Diseases 1996;28(6):924-30. [MEDLINE: 8957048]

Besarab 1998

Besarab A, Bolton WK, Browne JK, Egrie JC, Nissenson AR, Okamoto DM et al. The effects of normal, as compared with low, hematocrit values in patients with cardiac disease who are receiving hemodialysis and epoetin. New England Journal of Medicine 1998;339(9):584-90. [MEDLINE: 9718377]

Block 2004b

Block GA, Klassen PS, Lazarus LM, Ofsthun N, Lowrie EG, Chertow GM. Mineral metabolism, mortality and morbidity in maintenance hemodialysis. Journal of the American Society of Nephrology 2004;15(8):2208-10. [MEDLINE: 15284307]

Borrows 2004

Borrows R, Loucaidou M, Van Tromp J, Cairns T, Griffith M, Hakim N et al. Steroid sparing with tacrolimus and mycophenolate mofetil in renal transplantation. American Journal of Transplantation 2004;4(11):1845-51. [MEDLINE: 15476485]

Bucher 1999

Bucher HC, Guyatt GH, Cook DJ, Holbrook A, McAlister FA. Users' guides to the medical literature: XIX. Applying clinical trial results. A. How to use an article measuring the effect of an intervention on surrogate end points. Evidence-Based Medicine Working Group. JAMA 1999;282(8):771-8. [MEDLINE: 10463714]

CARI 2005

CARI Guidelines. http://www.kidney.org.au/cari/guidelines.php (accessed July 2005).

Churchill 1997

Churchill DN, Thorpe KE, Vonesh EF, Keshaviah PR. Lower probability of patient survival with continuous peritoneal dialyssi in the united states compared with canada. Canada-USA (CANUSA) Peritoneal dialysis study group. Journal of the American Society of Nephrology 1997;8(6):965-71. [MEDLINE: 9189865]

Courant 1993

Courant O, Letessier E, Moutel MG, Hamy A, Paineau J, Visset J. Surgical treatment of secondary hyperparathyroidism in chronic kidney failure. Results of total parathyroidectomy with parathyroid autotransplantation. Journal de Chirurgie 1993;130(8-9):327-34. [MEDLINE: 8253879]

Cunningham 2004

Cunningham J, Chertow G, Goodman W, Danese M, Olson K, Klassen P et al. The effect of cinacalcet HCl on parathyroidectomy, fracture, hospitalization and mortality in dialysis subjects with secondary hyperparathyroidism (HPT) [abstract]. In: 41st ERA-EDTA Congress; 2004; May 15-18; Lisbon (Portugal). 2004:219. [CENTRAL: CN-00509144]

Dickersin 1994

Dickersin K, Scherer R, Lefebvre C. Identifying relevant studies for systematic reviews. BMJ 1994;309(6964):1286-91. [MEDLINE: 7718048]

Egger 1997

Egger M, Davey-Smith G, Schneider M, Minder C. Bias in meta-analysis detected by a simple graphical test. BMJ 1997;315(7109):629-34. [MEDLINE: 9310563]

Egger 1998

Egger M, Smith GD. Bias in location and selection of studies. BMJ 1998;316(7124):61-6. [MEDLINE: 9451274]

FDA 2004

US Food and Drug Administration. Sensipar (cinacalcet HCl) tablets. http://www.fda.gov/cder/foi/label/2004/21688_Sensipar_lbl.pdf (accessed July 2006).

Ganesh 2001

Ganesh SK, Stack AG, Levin NW, Hulbert-Shearon T, Port FK. Association of elevated serum PO(4), Ca x PO(4) product, and parathyroid hormone with cardiac mortality risk in chronic hemodialysis patient. Journal of the American Society of Nephrology 2001;12(10):2131-8. [MEDLINE: 11562412]

Higgins 2003

Higgins JP, Thompson SG, Deeks JJ, Altman DG. Measuring inconsistency in meta-analyses. BMJ 2003;327(7414):557-60. [MEDLINE: 12958120]

Hutchison 1993

Hutchison AJ, Whitehouse RW, Boulton HF, Adams JE, Mawer EB, Freemont TJ et al. Correlation of bone histology with parathyroid hormone, vitamin D3, and radiology in end-stage renal disease. Kidney international 1995;26(5):622-31. [MEDLINE: 8264137]

Juni 2003

Juni P, Sterchi R, Dieppe P. Systematic review of celecoxib for osteoarthritis and rheumatoid arthritis. Problems compromise review's validity [letter]. BMJ 2003;326(7384):334. [MEDLINE: 12574052]

Kestenbaum 2005

Kestenbaum B, Sampson JN, Rudser KD, Patterson DJ, Seliger SL, Young B et al. Serum phosphate levels and mortality risk among people with chronic kidney disease. Journal of the American Society of Nephrology 2005;16(2):520-8. [MEDLINE: 15615819]

Lefebvre 1996

Lefebvre C, McDonald S. Development of a sensitive search strategy for reports of randomized controlled trials in EMBASE. In: Fourth International Cochrane Colloquium; 1996 Oct 20-24; Adelaide (Australia). 1996.

Leyland-Jones 2004

Leyland-Jones B. BEST Investigators and Study Group. Breast cancer trial with erythropoietin terminated unexpectedly. Lancet Oncology 2004;4(8):459-60. [MEDLINE: 12901958]

Malluche 2004b

Malluche HH, Mawad H, Monier-Faugere MC. The importance of bone health in end-stage renal disease: out of the frying pan, into the fire? Nephrology Dialysis Transplantation 2004;19 Suppl 1:i9-13. [MEDLINE: 15126648]

Marco 2003

Marco MP, Craver L, Betriu A, Belart M, Fibla J, Fernandez E. Higher impact of mineral metabolism on cardiovascular mortality in a European hemodialysis population. Kidney International - Supplement 2003;(85):S111-4. [MEDLINE: 12753279]

Martin 2004

Martin KJ, Olgaard K, Coburn JW, Coen GM, Fukagawa M, Langman C et al. Diagnosis, assessment and treatment of bone turnover abnormalities in renal osteodystrophy. American Journal of Kidney Diseases 2004;43(3):558-65. [MEDLINE: 14981615]

McMahon 2004

McMahon LP, Roger SD, Levin A. SLIMHEART Investigatos Group. Development, prevention and potential reversal of left ventricular hypertrophy in chronic kidney disease. Journal of the American Society of Nephrology 2004;15(6):1640-7. [MEDLINE: 15153576]

Moher 2001

Moher D, Schulz KF, Altman D CONSORT Group (Consolidated Standards of Reporting Trials). The CONSORT Statement: revised recommendations for improving the quality of reports of parallel-group randomized trials. JAMA 2001;285(15):1987-91. [MEDLINE: 11308435]

NKF 2003

National Kidney Foundation. K/DOQI clinical practice guidelines for bone metabolism and disease in chronic kidney disease. American Journal of Kidney Diseases 2003;42(4 Suppl 3):S1-210. [MEDLINE: 14520607]

Ott 1998

Ott SM. Calcimimetics--new drugs with the potential to control hyperparathyroidism. Journal of Clinical Endocrinology & Metabolism 1998;83(4):1080-2. [MEDLINE: 9543121]

Psaty 1999

Psaty BM, Weiss NS, Furberg CD, Koepsell TD, Siscovick DS, Rosendaal FR et al. Surrogate end points, health outcomes, and the drug-approval process for the treatment of risk factors for cardiovascular disease. JAMA 1999;282(8):786-90. [MEDLINE: 10463718]

Qi 1995

Qi Q, Monier-Faugere MC, Geng Z, Malluche HH. Predictive value of serum parathyroid hormone levels for bone turnover in patients on chronic maintenance dialysis. American Journal of Kidney Diseases 1995;26(4):622-31. [MEDLINE: 7573017]

Schulz 1995

Schulz KF, Chalmers I, Hayes RJ, Altman DG. Empirical evidence of bias. Dimensions of methodological quality associated with estimates of treatment effects in controlled trials. JAMA 1995;273(5):408-12. [MEDLINE: 7823387]

Sherrard 1998

Sherrard DJ. Calcimimetics in action. Kidney International 1998;53(2):510-1. [MEDLINE: 9461115]

Simes 1986

Simes RJ. Publication bias: the case for an international registry of clinical trials. Journal of Clinical Oncology 1986;4(10):1529-41. [MEDLINE: 3760920]

Stehman-Breen 2004

Stehman-Breen C. Osteoporosis and chronic kidney disease. Seminars in Nephrology 2004;24(1):78-81. [MEDLINE: 14730513]

Stevens 2004

Stevens LA, Djurdjev O, Cardew S, Cameron EC, Levin A. Calcium, phosphate, and parathyroid hormone levels in combination and as a function of dialysis duration predict mortality: evidence for the complexity of the association between mineral metabolism and outcomes. Journal of the American Society of Nephrology 2004;15(3):770-9. [MEDLINE: 14978180]

Temple 1999

Temple R. Are surrogate markers adequate to assess cardiovascular disease drugs? JAMA 1999;282(8):790-5. [MEDLINE: 10463719]

Urena 2003

Urena P, Frazao JM. Calcimimetic agents: review and perspectives. Kidney International - Supplement 2003;(85):S91-6. [MEDLINE: 12753275]

Wang 1995

Wang M, Hercz G, Sherrard DJ, Maloney NA, Segre GV, Pei Y. Relationship between intact 1-84 parathyroid hormone and bone histomorphometric parameters in dialysis patients without aluminum toxicity. American Journal of Kidney Diseases 1995;26(5):836-44. [MEDLINE: 7485142]

Young 2004

Young EW, Akiba T, Albert JM, McCarthy JT, Kerr PG, Mendelssohn DC et al. Magnitude and impact of abnormal mineral metabolism in hemodialysis patients in the dialysis outcomes and practice patterns study (DOPPS). American Journal of Kidney Diseases 2004;44(5 Suppl 3):34-8. [MEDLINE: 15486872]

Ziolkowska 2000

Ziolkowska H, Paniczyk-Tomaszewska M, Debinski A, Polowiec Z, Sawicki A, Sieniawska M. Bone biopsy results and serum bone turnever parameters in uremic children. Acta Paediatrica 2000;89(6):666-71. [MEDLINE: 10914959]

Other published versions of this review

Strippoli 2006

Strippoli GF, Palmer S, Tong A, Elder G, Messa P, Craig JC. Meta-analysis of biochemical and patient-level

effects of calcimimetic therapy. American Journal of Kidney Diseases 2006;47(5):715-26. [MEDLINE: 16632010]

Data and analyses

Figures

Figure 1

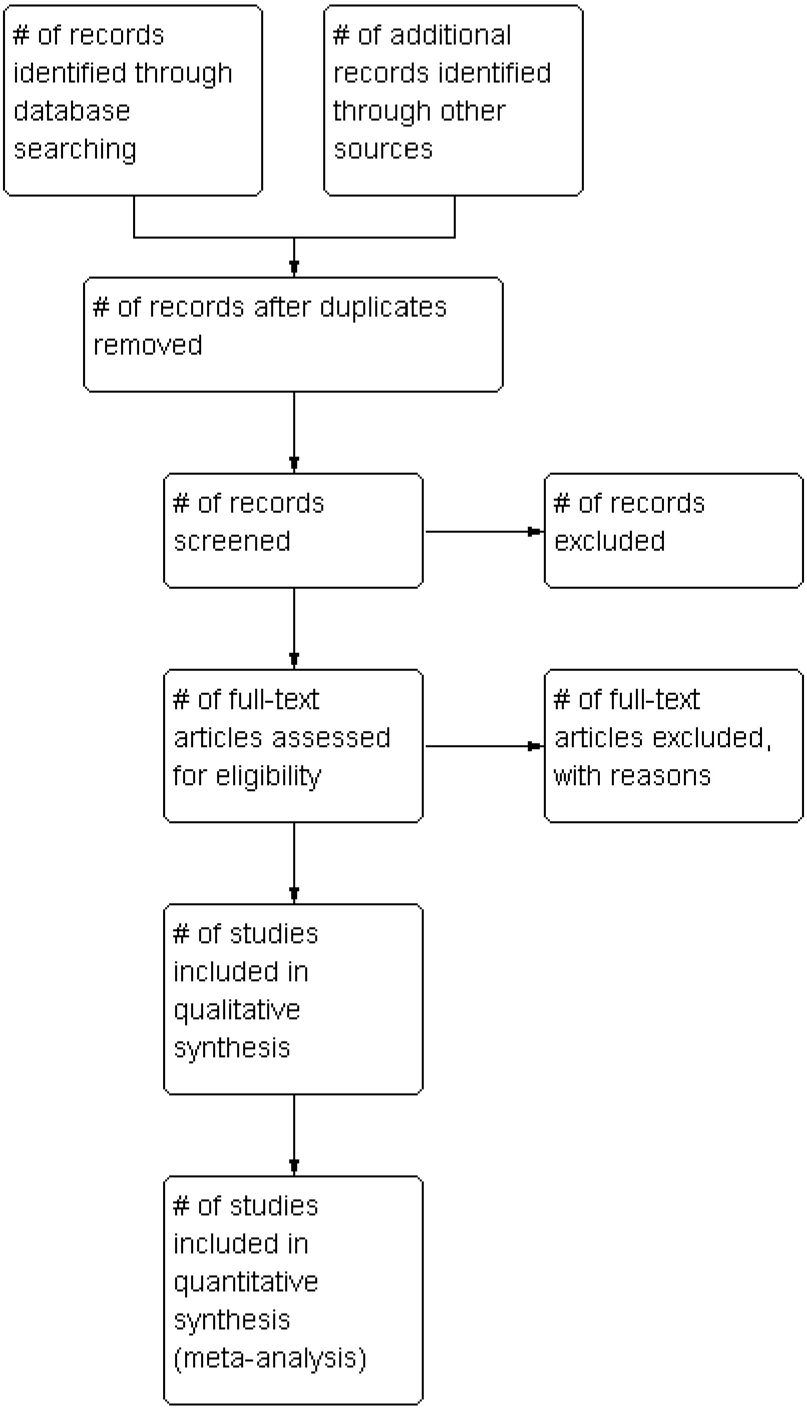

Study flow diagram.

Sources of support

Internal sources

- No sources of support provided

External sources

- No sources of support provided

Feedback

Appendices
